# Single-cell multiomic analysis of mesenchymal cells reveals molecular signatures and regulators of lung allograft fibrosis

**DOI:** 10.1172/jci.insight.196651

**Published:** 2026-04-23

**Authors:** Lu Lu, A. Patrick McLinden, Natalie M. Walker, Ragini Vittal, Yichen Wang, Fatemeh Fattahi, Stephen T. Russell, Michael P. Combs, Joshua D. Welch, Vibha N. Lama

**Affiliations:** 1 Department of Computational Medicine and Bioinformatics, University of Michigan, Ann Arbor, Michigan, USA.; 2 Division of Pulmonary, Allergy, Critical Care, and Sleep Medicine, School of Medicine, Emory University, Atlanta, Georgia, USA.; 3 Division of Pulmonary & Critical Care, School of Medicine, and; 4 Department of Computer Science and Engineering, University of Michigan, Ann Arbor, Michigan, USA.

**Keywords:** Genetics, Pulmonology, Fibrosis, Organ transplantation

## Abstract

Survival after lung transplantation is limited by chronic, progressive graft failure, termed chronic lung allograft dysfunction (CLAD). Graft-resident mesenchymal cells (MCs) drive CLAD pathogenesis and exhibit stable dysregulated signaling, yet the transcriptomic and epigenomic drivers underlying this fibrogenic transformation remain elusive. We used single-cell multiomic profiling to characterize gene expression and chromatin accessibility in MCs isolated from bronchoalveolar lavage fluid of lung transplant recipients with and without CLAD, collected early after transplantation or after disease onset. MCs obtained after CLAD onset demonstrated a distinct transcriptomic signature compared with non-CLAD controls, enabling classification of disease status at the single-cell level with greater than 98% accuracy using signature genes. Chromatin accessibility analyses identified enrichment of CCAAT-enhancer-binding protein family transcription factors, specifically CEBPD, in CLAD MCs. MCs early after transplantation showed minimal accessibility differences, suggesting that CEBPD-associated regulatory changes emerge over time. Integration analyses identified 8 MC states and a CLAD-specific shift toward a fibrotic state. CEBPD, SOX4, and FOXP2 were identified as putative regulators of this state with substantial overlap in predicted targets. Targeting CEBPD reversed fibrotic phenotypes of CLAD MCs (decreased ECM expression, contractility, proliferation, and migration). Together, these data provide insights into transcriptomic and epigenomic changes in posttransplant MCs, facilitating the nomination of biomarkers and therapeutic targets.

## Abstract 2

Single-cell multi-omic profiling of cultured mesenchymal cells reveals CEBPD as a key regulator of a fibrotic cell state in chronic lung allograft dysfunction.

## Introduction

Mesenchymal cell (MC) infiltration and matrix deposition leading to architectural distortion are key features of fibrotic diseases across all organs (1). Considerable insight has been gained into the mechanisms of fibrosis by in vitro investigations of MCs derived from diseased human tissues. While initial focus was on mediators present in the profibrotic milieu that induce and sustain myofibroblast differentiation (2, 3), an emerging paradigm of an autonomous fibrotic transformation of MCs has recently emerged with demonstration of a myriad of stable changes at the transcriptional and translational level in these MCs removed from their microenvironment (4–8). Recent advancements in single-cell sequencing technologies provide a unique opportunity to improve upon this work by delving into the intricate heterogeneity of cells in culture and studying genetic and epigenetic molecular signatures in an unbiased manner.

Lung transplantation, the only viable option available to patients with end-stage lung diseases, presents a formidable challenge with a median survival of 5.8 years, the lowest among all solid organ transplants (9). The major cause of these poor long-term outcomes is chronic lung allograft dysfunction (CLAD), characterized by recalcitrant fibrotic remodeling of the allograft and progressive lung function decline (10). CLAD develops in greater than 50% of patients by 5 years of transplantation and is the major cause of mortality 1 year after lung transplantation (10). Allograft fibrosis, emanating from the bronchovascular bundles and small airway fibrosis or bronchiolitis obliterans, is a key feature of the failing grafts in CLAD. Therapeutic approaches to modulate allograft fibrogenesis are a key unmet need in this arena, as augmented immunosuppression fails to alter the progression of chronic graft failure meaningfully.

A substantial body of translational work from our laboratory has highlighted a stable alteration in graft-resident MCs that perpetuates fibrosis in a chronically rejecting lung allograft. We identified a resident population of mesenchymal progenitors in human adult lungs by demonstrating that MCs isolated from bronchoalveolar lavage (BAL) of lung allografts are of donor origin and retain the expression of embryonic lung mesenchyme-associated transcription factor Forkhead box gene *FOXF1* (11, 12). This transcriptionally distinct *Foxf1*^+^ subset of collagen I–expressing lung-resident MCs was shown to reside topographically along the bronchovascular bundles and contribute to allograft fibrosis (12, 13). Translational human studies demonstrated that their expansion (14, 15) and fibrotic differentiation (6, 7, 16–18) precede and accompany the development of CLAD. Mechanistic investigations have revealed stable dysregulation across a myriad of signaling pathways in human CLAD lung–derived MCs, suggesting a yet undiscovered alteration in global transcriptional and epigenomic landscape (6, 7, 11, 13, 16–18). Whether the altered fibrogenic state of these graft-resident MCs is acquired over time after transplantation also remains to be investigated.

In this study, we leverage single-cell multiomics profiling and provide an unbiased examination of distinct MC gene expression and chromatin accessibility signatures in CLAD. Our findings elucidate the molecular signatures associated with CLAD and highlight temporal changes in human lung allograft–derived MCs marked by unique gene expression profiles, regulatory networks, and epigenetic modifications. We nominate putative regulators of the dysregulated molecular states of CLAD MCs and specifically highlight a central role for transcription factor (TF) CCAAT/enhancer-binding protein delta (CEBPD) in sustaining the fibrotic state of MCs. Additional single-cell RNA sequencing (scRNA-seq) analysis of MCs from human CLAD and normal lungs further demonstrated that cultured MCs retain and reflect key transcriptomic signatures from their in vivo counterparts.

## Results

### Single-cell multiomic profiling of human MCs from lung transplant recipients.

**Figure 1:**
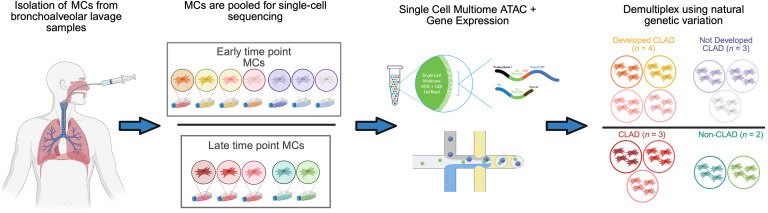
Experimental strategy. Mesenchymal cells (MCs) were isolated from bronchoalveolar lavage (BAL) fluid derived from lung transplant recipients. MCs were screened using clinical parameters and then pooled together prior to single-cell multiome profiling. The donor identity of each cell was determined using genetic variants captured in the gene expression and chromatin accessibility reads. Workflow was created using BioRender.com.

To investigate whether CLAD is associated with stable transcriptomic and epigenomic changes in graft MCs, joint scRNA-seq and single-cell assay for transposase-accessible chromatin using sequencing (scATAC-seq) was performed on MCs isolated from BAL of lung transplant recipients (Figure 1 and Supplemental Table 1; supplemental material available online with this article; https://doi.org/10.1172/jci.insight.196651DS1). MCs from patients who had a diagnosis of CLAD at the time of BAL (CLAD MCs) were compared to the MCs obtained from control CLAD-free patients (non-CLAD MCs). The incidence of CLAD increases with time after transplantation, and CLAD diagnosis is generally made after the first year of lung transplantation. To investigate whether transcriptional and epigenomic changes are inherent or acquired, MCs were also studied from BAL samples obtained early in the course (<6 weeks after transplantation). These patients were later clinically classified as having developed CLAD or remained CLAD-free. Single-cell multiome sequencing with the 10X Genomics Chromium platform was performed. To lower library preparation costs, MCs were pooled before loading them onto the 10X Chromium, allowing profiling of multiple donors simultaneously. Each donor was genotyped using an Illumina SNP array. scRNA-seq and scATAC-seq reads were demultiplexed using this genotype information (Figure 1 and Supplemental Figure 5; see Methods and Supplemental Methods). After quality control filtering, we obtained 22,918 MCs in total for further analysis (Supplemental Figure 1).

### Classification models predict CLAD status of patient-derived MCs from single-cell gene expression.

**Figure 2:**
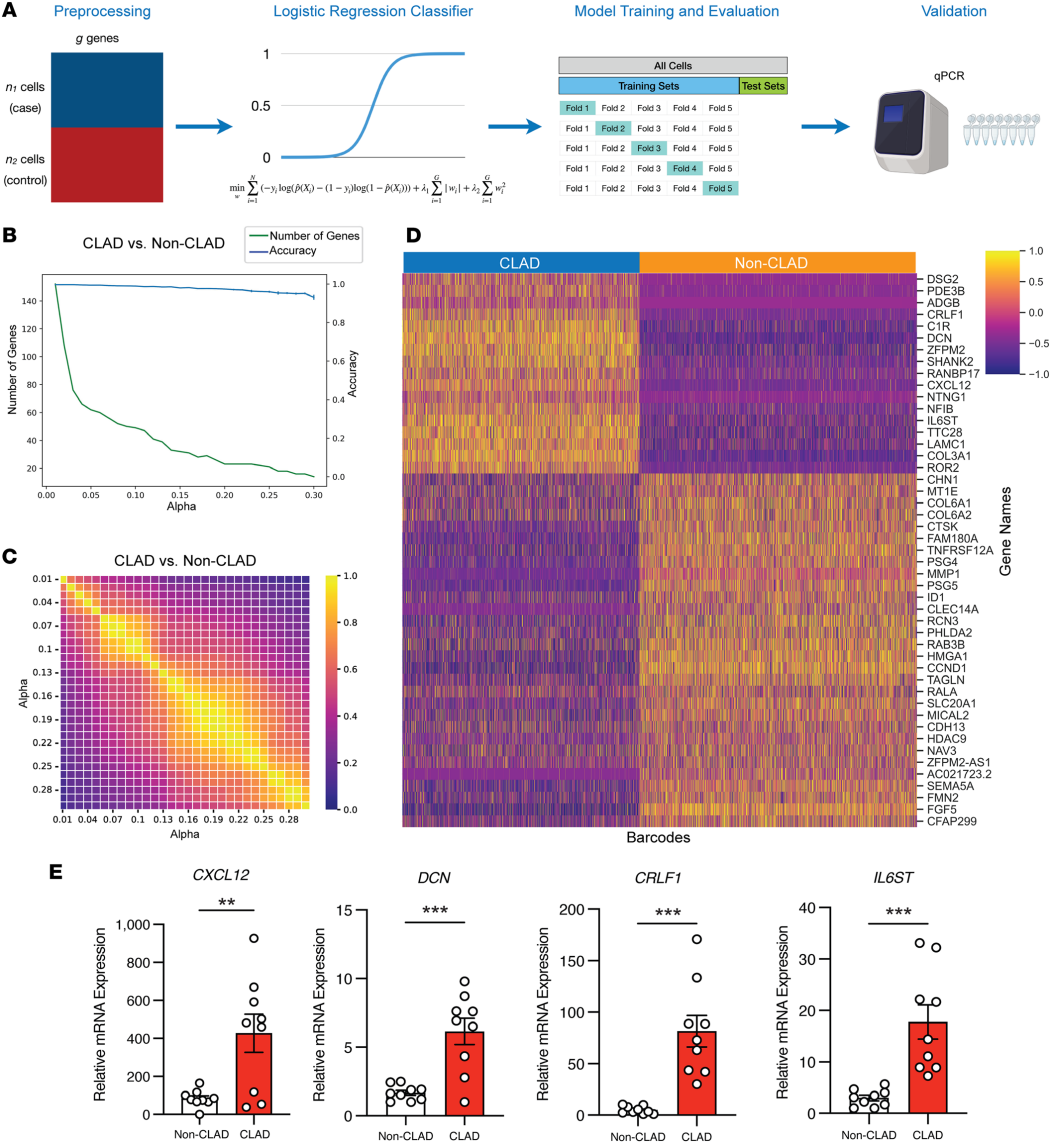
Classifying CLAD status from single-cell gene expression. (**A**) Workflow of training logistic regression classifier. (**B**) The blue line indicates average accuracy score of the logistic regression classifiers from 5-fold cross-validation. Error bars indicate the variance in accuracy across folds. The green lines indicate the number of genes with non-zero coefficients from the logistic regression classifiers trained on the test sets. (**C**) Heatmap showing the overlap of genes among different coefficients. (**D**) Heatmaps depicting the expression levels of gene signatures in CLAD patient–derived MCs constructed from the logistic regression classifiers with α of 0.1. Each column represents a cell, and each row represents 1 gene colored by gene expression level. (**E**) RT-qPCR analysis of *CXCL12*, *DCN*, *CRLF1*, and *IL6ST* expression from CLAD and non-CLAD cell lines. **P ≤ 0.01; ***P ≤ 0.001 by unpaired, 2-tailed Student’s *t* test.

To identify transcriptomic markers of CLAD in patient-derived MCs, we trained classification models to distinguish cells originating from CLAD versus non-CLAD lungs (Figure 2A). To determine the optimal number of genes in the classifier, we quantified the model performance while varying the parameter, which controls the number of genes with nonzero regression coefficients. Higher values of indicate a stronger regularization and result in fewer genes selected. The accuracy score and the corresponding number of genes for each α-value were reported (Figure 2B). The classifiers achieved high accuracy, with the highest test accuracy of 99.2% on classifying the late-time-point MCs (Figure 2B). In addition, we calculated the Jaccard similarity index between each set of selected genes across different parameter values (Figure 2C). The high similarity score between adjacent pairs of α values indicates that a key set of genes persists across a range of α parameters. To ensure that our model learned the true biological signal of CLAD status, rather than simply biological variance among donors, we additionally evaluated our model by holding out all cells from one donor at a time. We observed similar performance training on all donors compared to one donor leave-out, indicating that the model is not simply capturing biological variation among donors (Supplemental Figure 2).

When applying the same classification approach to the early-time-point MCs, we found that classification was more challenging compared with the late-time-point MCs. The highest test accuracy achieved was 91.2%, and the classifier required more gene features to attain this performance (Supplemental Figure 3A). This suggests that there is less transcriptional difference among the early-time-point MCs than the late-time-point MCs. Differential expression analysis further supports this observation, in that only 66 genes were significantly differentially expressed in the early-time-point MCs. In comparison, 1,707 genes were significantly differentially expressed in the late-time-point MCs (Supplemental Figure 3B).

To select a final α value for subsequent analyses, we identified an “elbow” in the plot of accuracy vs number of genes, choosing a model that uses as few genes as possible without a notable drop in accuracy. We opted for an α of 0.1, which identified 47 genes for the late-time-point group (Figure 2D), and an α of 0.05, resulting in the selection of 54 genes for the early-time-point group (Supplemental Figure 3C). These classifiers achieved test accuracy of 98.7% and 85.8% in the late- and early-time-point groups, respectively. Many of the genes selected by these classifiers are plausibly linked to CLAD based on previous studies. The selected genes for CLAD include *DCN*, a known gene involved in fibrogenesis (19). We observed that *CXCL12*, an important homeostatic chemokine implicated in both homeostatic and inflammatory contexts (20–24), was upregulated in CLAD MCs (Figure 2D). To verify differential expression of select genes discovered through scRNA-seq of CLAD MCs, we performed real-time quantitative PCR (RT-qPCR) for *CXCL12*, *DCN*, *CRLF1*, and *IL6ST* in MCs isolated from additional CLAD and non-CLAD donors (Figure 2E). We successfully verified increased mRNA expression in CLAD MCs for each of the 4 genes examined (Figure 2E).

### Chromatin accessibility analysis highlights CEBPD as a key positive regulator in CLAD patient–derived MCs.

**Figure 3:**
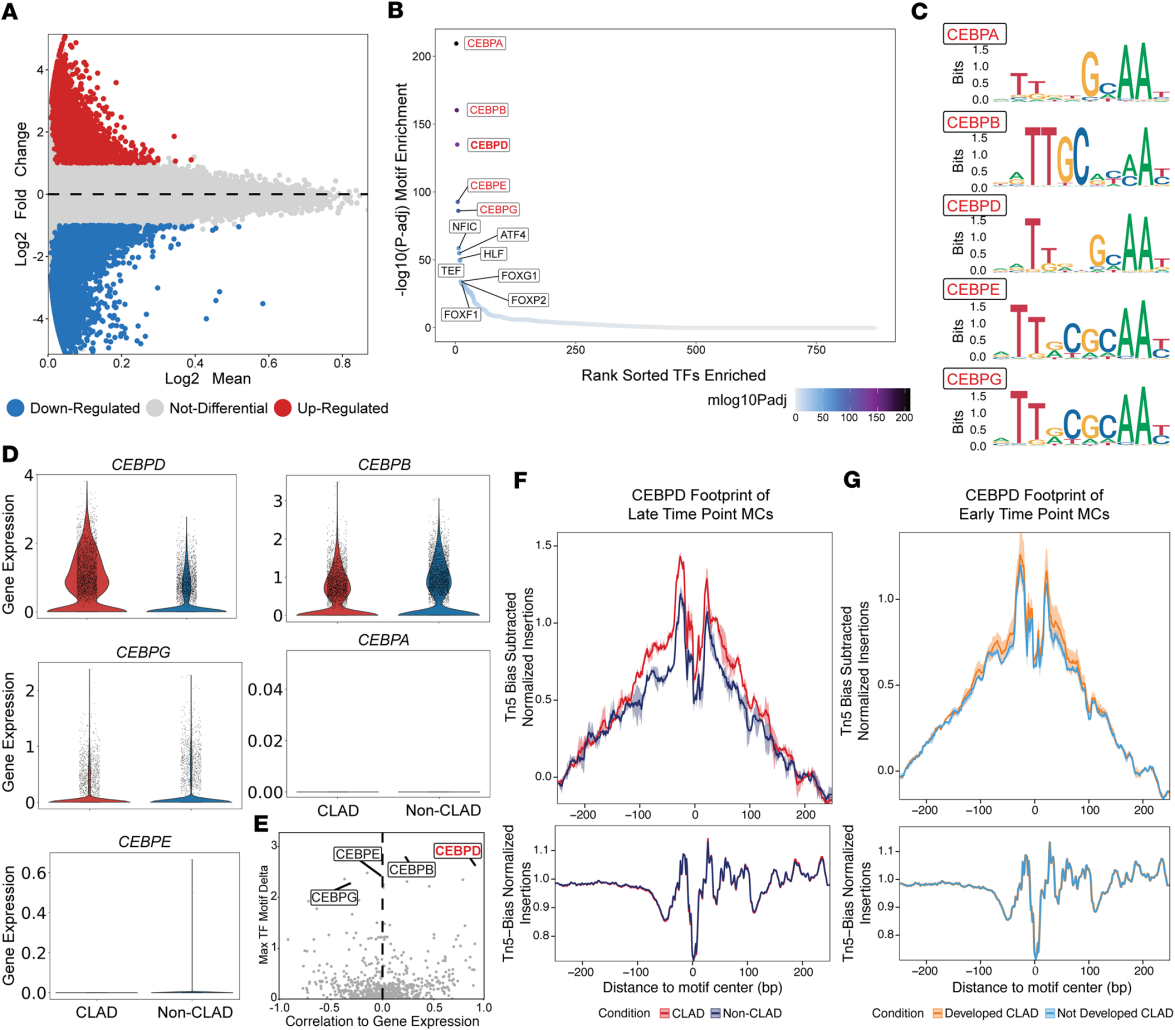
Single-cell chromatin accessibility analysis to identify epigenetic markers of CLAD MCs. (**A**) MA plot showing marker peaks in CLAD. (**B**) TF motifs enriched in CLAD ranked by enrichment. (**C**) Sequence logo of motifs from CEBP family members (CEBPA, CEBPB, CEBPD, CEBPE, and CEBPG) in position weight matrices (PWMs). (**D**) Violin plots depicting the expression levels of CEBP family members (CEBPA, CEBPB, CEBPD, CEBPE, and CEBPG) across conditions. The width of each plot represents the distribution of expression values. Comparisons are shown between late-time-point MCs with (red) or without (blue) CLAD. (**E**) Dot plot showing correlation between motif accessibility and its corresponding TF expression. The *x*-axis is Pearson’s correlation coefficient between motif deviation *z* scores from chromVAR and its corresponding TF expression. The *y*-axis is the TF deviation *z* scores from chromVAR. TFs from CEBP family memebrs are annotated and CEBPD is highlighted in red. (**F** and **G**) CEBPD footprinting of late-time-point (**F**) and early-time-point (**G**) MCs.

We next examined differences in chromatin accessibility profiles between CLAD and non-CLAD MCs. First, we performed peak calling on each sample, then merged the peaks, identifying 114,464 accessible peaks that were concordant among donors. Quality control to filter cells was performed for each sample separately (see Methods and Supplemental Figure 4). To find peaks with differential accessibility in each condition, we performed Wilcoxon rank-sum tests on CLAD and non-CLAD samples. Among 114,464 accessible peaks, we identified 2,983 (2.61%) upregulated peaks and 4,120 (3.60%) downregulated peaks in CLAD by setting filtering thresholds of an FDR of 0.05 or less and an absolute log_2_(fold change) of 1 or greater (Figure 3A).

To understand the key TFs that could bind to these accessible chromatin sites, we then performed a motif scan. The CCAAT-enhancer-binding protein (CEBP) family of transcription factors, including CEBPD, a TF involved in proliferation, apoptosis, and possibly fibrogenesis (25–27), was noted to be enriched in CLAD (Figure 3B). However, members of the CEBP family share high similarities in their binding motifs, which can be visualized as sequence logos derived from position weight matrices (PWMs) (Figure 3C) (28). This motif similarity is common among many TF families, making it challenging to pinpoint the specific TFs that might be involved in regulation (29). To address this, we incorporated the matched gene expression data. We first examined the expression of the CEBP family and found that CEBPD is the only TF among the CEBP family that is enriched in CLAD; it exhibited 32-fold higher expression in CLAD MCs than in non-CLAD MCs (Figure 3D). CEBPB showed similar expression levels between the two conditions, while CEBPA, CEBPE, and CEBPG were either expressed at low levels or not expressed at all (Figure 3D). We further calculated a Pearson correlation between the gene expression of TFs and the chromatin accessibility of their corresponding motif. CEBPD yielded the highest correlation of 0.9 (adjusted *P* = 2.43 × 10^–179^) (Figure 3E). Moreover, we performed footprinting analysis at predicted CEBPD binding sites. When a TF is bound to DNA, it physically protects that region from transposase activity during ATAC-seq, creating a characteristic footprint, or a localized dip in accessibility at the center of the motif, flanked by regions of higher accessibility. This footprint pattern was clearly observed at CEBPD motifs, indicating active binding of CEBPD (Figure 3F). The footprint pattern exists in both conditions, but the significant difference in accessibility suggests a change in binding affinity between CLAD and non-CLAD MCs, which may drive the gene regulatory differences. In contrast, footprint analysis of early-time-point MCs revealed minimal differences in accessibility, suggesting that CEBPD-associated regulatory changes emerge later in the disease course and are not present shortly after transplantation (Figure 3G). Taken together, these results point to CEBPD as a candidate TF driving gene regulatory changes in CLAD MCs and such alterations do not take place early in the transplantation.

### Single-cell analysis reveals temporal cell state shifts in patient-derived MCs.

**Figure 4:**
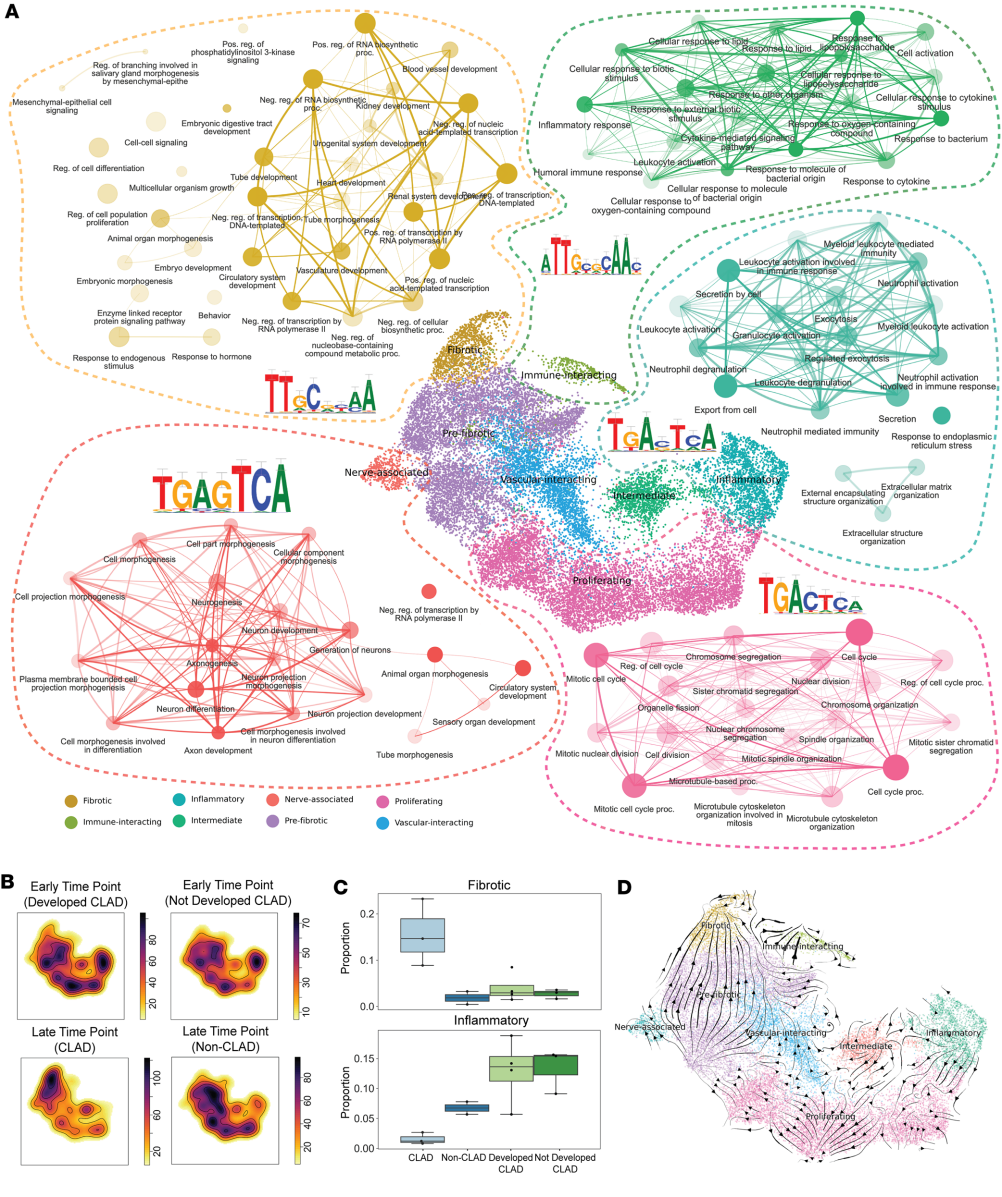
Cell state heterogeneity and cell state shifts in patient-derived MCs. (**A**) Uniform manifold approximation and projection (UMAP) representation of 22,918 cells derived from lung transplant recipients at the 2 time points. Each dot represents a single cell, and cells are colored by clusters. (**B**) Density plots of integrated MCs. Top left: Early time point (later developed CLAD). Top right: Early time point (later not developed CLAD). Bottom left: Late time point (CLAD patient–derived). Bottom right: Late time point (non–CLAD patient–derived). (**C**) Compositional change in fibrotic (top) and inflammatory (bottom) clusters. The bounds of the boxes represent the 25th and 75th percentiles (interquartile range, IQR); the line within each box represents the median; the whiskers extend to the minimum and maximum values within 1.5× IQR from the box bounds; individual data points beyond the whiskers are plotted as outliers. (**D**) Velocities fit by VeloVAE are visualized as streamlines in the UMAP plot.

To explore the compositional heterogeneity in MCs derived from lung transplant patients across different time points, we performed integration and unsupervised clustering using linked inference of genomic experimental relationships (LIGER) (30, 31). We integrated samples from 12 donors to identify shared and distinct cell states between CLAD and control cells despite biological variation among donors (Figure 4, A and B). We then annotated clusters using top Gene Ontology (GO) terms from differentially expressed genes (DEGs) and enriched TF motifs (Figure 4A). We also compared published marker genes from previously identified fibroblast cell states, and these published studies were utilized to help with the nomenclature (32, 33). Eight distinct cell states were identified and annotated as proliferating, nerve-associated, immune-interacting, vascular-interacting, intermediate, prefibrotic, fibrotic, and inflammatory (Figure 4A). The marker genes and GO terms for the vascular-interacting and immune-interacting populations are concordant with those identified in a previous cross-tissue study of fibroblast cell states (32). Immune-interacting clusters are enriched for GO terms related to humoral immune response (34) and cytokine-mediated signaling. The nerve-associated cluster exhibits GO terms associated with neurogenesis and neuronal development. The features present in this cluster are similar to those of recently discovered fibroblasts related to airway peripheral nerves (33). The proliferating cell state is distinguished largely by expression of mitotic cell cycle genes, microtubule and cytoskeleton genes, and other markers of proliferation. GO terms enriched in the fibrotic cluster included positive regulation of transcription by RNA polymerase II, cell surface receptor signaling pathway, regulation of cell differentiation, cell communication, signal transduction, and cell motility. These included genes such as *CRLF1*, *VEGFA*, *HDAC4*, *ITGB8*, and *CXCL12*. Molecular processes identified by GO analysis in this cluster included terms such as CD4 receptor binding and extracellular matrix (ECM) structural constituents with genes such as *IL16*, *PLSCR4*, *PLSCR1*, various collagens, *MRXA5*, and *LAMB1*. The inflammatory cluster shows upregulation of neutrophil-mediated immunity and neutrophil activation genes involved in immune responses.

Having characterized the cell state diversity of the MCs, we next looked for cell state shifts/compositional differences between MCs across different conditions (Figure 4B). A striking observation was the unique pattern that emerged for CLAD MCs where there was a shift toward the fibrotic state. Most of the cells in the fibrotic cluster came from CLAD patients (Figure 4C). The inflammatory state was predominantly enriched in early-time-point MCs, possibly reflecting innate immune engagement during the early posttransplant period. Notably, the composition of early-time-point MCs was highly similar between patients who later developed CLAD and those who did not develop CLAD, with MCs collected from patients who later developed CLAD having a slight shift toward the fibrotic state. These findings are consistent with previous observations that transcriptional and epigenetic differences between CLAD and non-CLAD MCs emerge later in the disease course and are not apparent shortly after transplantation. Collectively, these results indicate that MCs from different time points and conditions occupy different parts of the fibroblast cell state space.

RNA velocity analysis using VeloVAE (35) to investigate potential relationships among the cell states was performed (Figure 4D). The primary cellular state transition suggested was from prefibrotic to fibrotic, with no clear unidirectional relationship between other states.

### Enhancer-driven gene regulatory network analysis reveals putative regulators of the CLAD-enriched fibrotic state.

**Figure 5:**
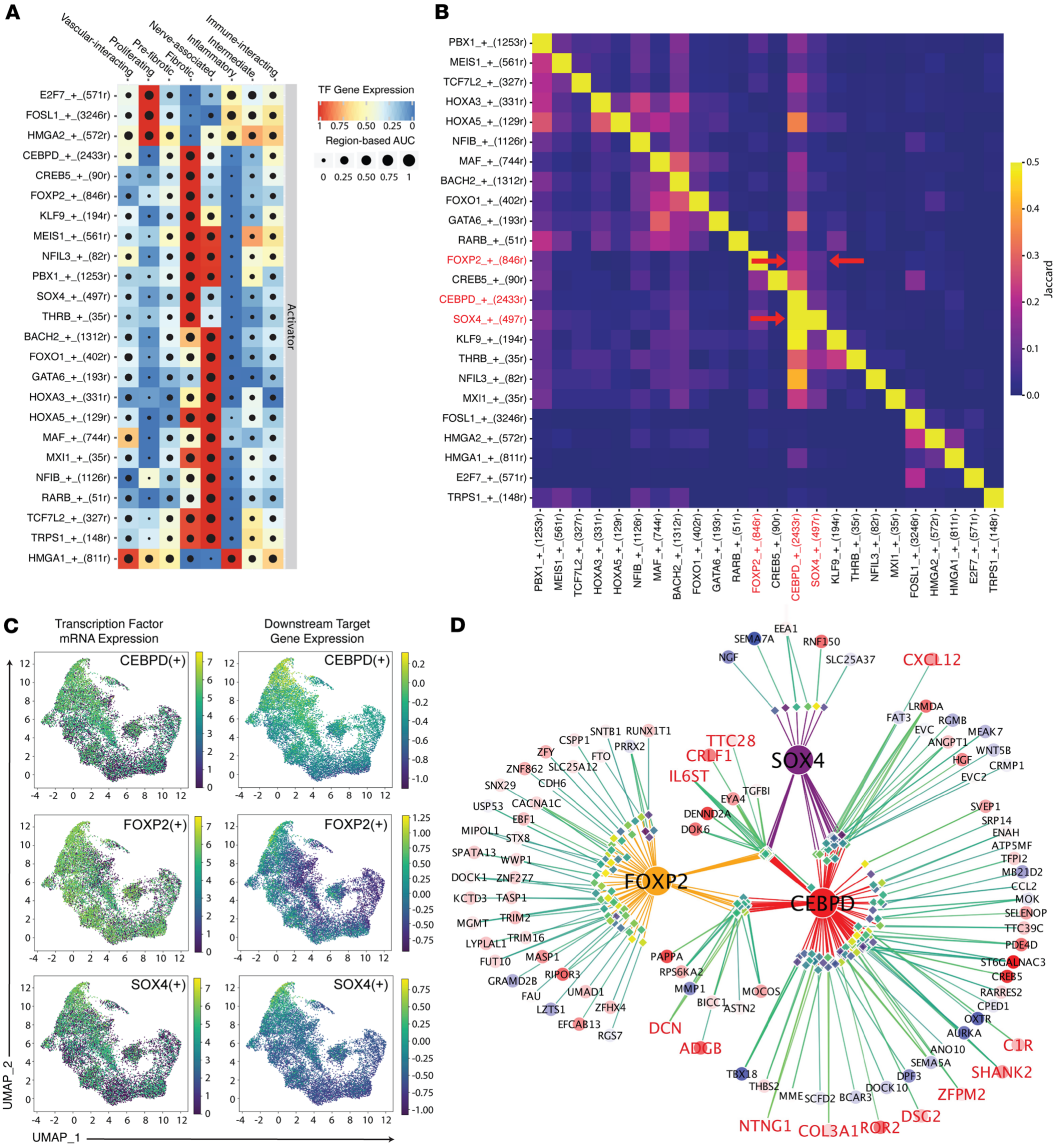
Gene regulatory analysis combining single-cell transcriptomic and epigenomic features nominates regulators of CLAD-enriched fibrotic state. (**A**) Dot plot showing the state specificity of the predicted targets for each TF. Each row is a TF and each column is a cell state. The color of each square indicates the aggregate expression of predicted target genes for each TF in each cell state. Dot size indicates the area under the recovery curve (AUC) for a classifier trained to predict the cell state from the chromatin accessibility of target peaks. Higher AUC means that the target regions for the TF are more state-specific. (**B**) Heatmap showing overlap between predicted target regions of each TF. Each square shows the Jaccard similarity index (intersection of target regions divided by the union of target regions) for the pair of TFs in the corresponding row and column. Higher values indicate more overlap between the targets of the pair of TFs. (**C**) UMAP visualization of CEBPD, FOXP2, and SOX4 and predicted targets. Each dot represents a cell. Dots are colored by TF expression (left) or the sum of target gene expression (right). (**D**) Visualization of the enhancer-driven gene regulatory network formed by CEBPD, FOXP2, and SOX4. Each circle is a gene; color and size represent the log_2_(fold change) of the gene’s expression in fibrotic MCs compared with the rest of the cells. Thus, a large, red circle indicates a gene with much higher expression in fibrotic MCs and a large blue circle indicates a gene with much lower expression in fibrotic MCs. Each diamond is a chromatin accessibility peak (putative enhancer) and its color represents the log_2_(fold change) of the accessibility in fibrotic MCs compared with the rest of the cells (yellow is higher accessibility in fibrotic MCs). Each line indicates a regulatory relationship; line color indicates the strength of the correlation between peak and gene (higher correlation is more yellow). Gene symbols in red letters are part of the CLAD signature from Figure 2E.

The joint gene expression and chromatin accessibility data we collected provided a unique opportunity to investigate regulatory activity involving the expression of TFs, the chromatin accessibility of TF-binding sites, and the expression of target genes within MCs among different cellular states. For this purpose, we used SCENIC^+^ to infer an enhancer-driven gene regulatory network (eGRN) (36). SCENIC^+^ looks for 3-way relationships in which TFs are expressed in cells where peaks containing binding sites for the TFs are accessible, and the accessibility of the binding sites is also correlated with the expression of nearby genes. In total, this analysis identified 447 TFs that are predicted to regulate downstream target genes. Among these TFs, we identified 24 activator eRegulons (correlation coefficient > 0.5) predicted to target a total of 12,174 regions and 3,703 target genes. We also calculated the cell state specificity of each activating TF (Figure 5A). For example, one notable activating factor is NFIB, a TF known to be expressed in lung MCs (37). NFIB shows the highest activity in the nerve-associated state (Figure 5A). Research has demonstrated that NFIB plays an important role in the development and progression of various nerve-related diseases (38, 39). Moreover, HMGA1, a regulator enriched in the inflammatory cluster, has been shown to be involved in inflammatory pathways (40). This is consistent with our observation that, early after lung transplantation, MCs present an inflammatory state. CEBPD, the transcription factor found to be enriched in the CLAD state, showed the highest activity in the fibrotic cluster.

We further examined activating factors enriched in the fibrotic cell state. The factors with the most state-specific expression in the fibrotic state and the most predicted targets are CEBPD (2,433 targets), FOXP2 (846 targets), and SOX4 (497 targets). Although PBX1 (1,253 targets) and MEIS1 (561 targets) also have high expression in the fibrotic state and a substantial number of predicted targets, they additionally show substantial activity in the nerve-associated state, indicating that their activity is less specific to the fibrotic state. Interestingly, the predicted targets of CEBPD, FOXP2, and SOX4 substantially overlap, while PBX1 and MEIS1 are predicted to regulate a separate set of genes that overlap between the two factors but are largely disjoint from genes regulated by CEBPD, FOXP2, and SOX4 (Figure 5B). The aggregate expression of predicted gene targets of CEBPD, FOXP2, and SOX4 shows strong enrichment in the fibrotic state (Figure 5C). Strikingly, this aggregate expression is more state-specific than the gene expression of the TFs themselves, likely reflecting state-specific differences in the chromatin accessibility of the TF binding motifs.

We further investigated the predicted gene and peak targets of CEBPD, FOXP2, and SOX4. We plotted their gene regulatory network to confirm the observation of cooperativity among and visualize the target genes of the factors (Figure 5D). Interestingly, 13 of these target genes were also predicted by our logistic regression model as key upregulated genes related to CLAD (Figure 2E). Several of the CLAD signature genes, including *DCN*, *CXCL12*, *IL6ST*, *CRLF1*, *TTC28*, and *ADGB*, are also predicted to be co-regulated by 2 or more of the factors.

### CEBPD knockdown partially reverts fibrotic gene expression signatures.

**Figure 6:**
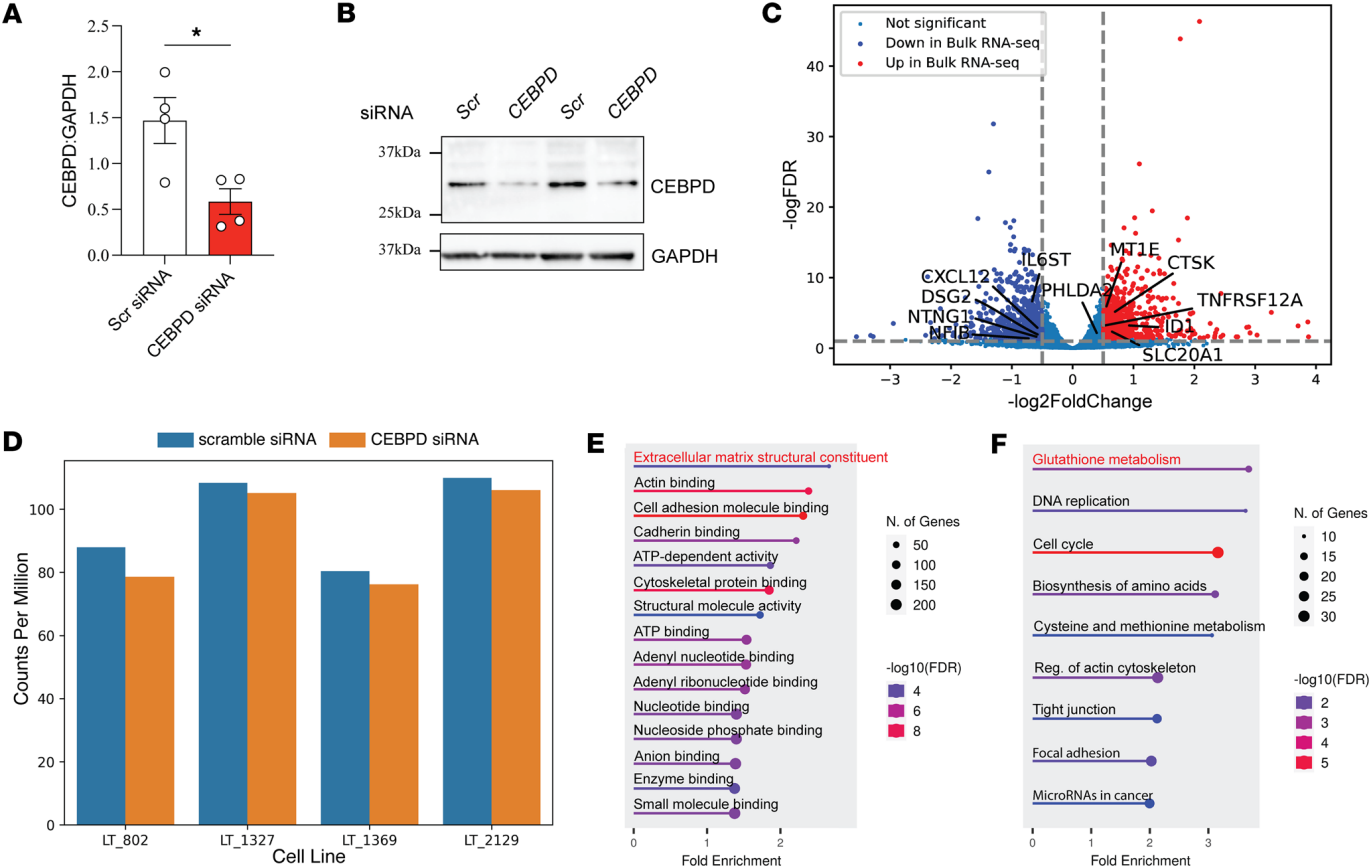
*CEBPD* knockdown partially reverts CLAD gene expression signature. (**A**) RT-qPCR analysis of expression of *CEBPD* relative to GAPDH in CLAD MCs treated with scrambled or *CEBPD* siRNA. Comparisons were conducted using unpaired, 2-tailed Student’s *t* test. *P < 0.05. (**B**) Protein expression of CEBPD in CLAD MCs treated with scrambled or *CEBPD* siRNA as measured by Western blot analysis. (**C**) Volcano plot illustrating the differentially expressed genes following *CEBPD* knockdown in CLAD MCs. The *x*-axis represents the log_2_(fold change), and the *y*-axis represents the –logFDR. Genes that are significantly upregulated (log_2_FC > 0.5, adj. *P* < 0.05) are marked in red, while significantly downregulated genes (log_2_FC < –0.5, adj. *P* < 0.05) are marked in blue. Non-significant genes are shown in gray. Gene symbols highlighted with texts are part of the CLAD signature from Figure 2E. (**D**) Bar plot showing the total expression of the upregulated CLAD signature genes between CLAD MCs treated with scrambled (blue) or *CEBPD* (orange) siRNA. The *x*-axis is the cell line; control and knockdown samples from the same line are shown as adjacent bars. The *y*-axis represents the log sum of counts per million. (**E**) GO enrichment analysis showing downregulation of ECM-related genes after *CEBPD* knockdown. (**F**) KEGG pathway enrichment analysis showing downregulation of glutathione metabolism–related genes after *CEBPD* knockdown.

Given that CEBPD is noted to be enriched in the CLAD disease state and is the fibrotic cluster-specific factor with the most predicted targets (and the most predicted targets that are also part of our CLAD signature), we decided to further explore the regulatory role of CEBPD. To do this, we knocked down *CEBPD* using small interfering RNAs (siRNAs). CLAD MCs were treated with either a *CEBPD*-targeting siRNA (treated group) or a scrambled siRNA (control group) for 16 hours. Knockdown efficacy was confirmed at both the mRNA and protein levels using qPCR and Western blots (Figure 6, A and B). To measure the gene expression changes induced by *CEBPD* silencing, we performed bulk RNA-seq on both the treated and control groups across the 4 CLAD samples. After sequencing, we obtained more than 36 million reads per sample mapped to genes. Differential expression analysis revealed a total of 2,436 DEGs between control and knockdown samples, with 1,265 genes upregulated and 1,171 genes downregulated after *CEBPD* silencing (Figure 6C). The overlap between DEGs from the bulk RNA-seq and the predicted downstream targets of CEBPD from the eGRN was examined. Of the 593 genes predicted to be downstream targets of CEBPD as an activator, 85 were indeed downregulated after *CEBPD* silencing, while 64 remained upregulated (Supplemental Figure 6A). Additionally, of the 201 genes predicted to be downstream targets of CEBPD as a repressor, 11 were upregulated and 32 remained downregulated. Next, we compared the DEGs from the bulk RNA-seq with the previously identified CLAD signature in Figure 2 from the logistic regression model (Supplemental Figure 6B). None of the upregulated genes in CLAD showed significantly higher expression in the *CEBPD*-silenced group than in the control group. Notably, 5 of the 17 upregulated genes in CLAD, including *CXCL12* and *IL6ST*, were significantly downregulated in the bulk RNA-seq (Figure 6C). Similarly, 6 out of 30 downregulated genes in CLAD were significantly upregulated in the bulk RNA-seq after knockdown (Figure 6C). Furthermore, we added up the total expression of all 17 upregulated signature genes in both control and knockdown cells, and found that the total expression of the upregulated CLAD signature genes (in units of counts per million) was lower in each of the 4 knockdown samples compared with its donor-matched control sample (Figure 6D). These results suggest that silencing *CEBPD* partially reverts the CLAD gene expression signature we identified.

To further understand the transcriptomic changes induced by CEBPD knockdown, we performed GO enrichment analysis and examined enriched Kyoto Encyclopedia of Genes and Genomes (KEGG) pathways (Figure 6, E and F). The top GO terms identified as downregulated were ECM structural constituent and actin binding, with decreased expression of genes such as *COL11A1*, *COL12A1*, *FBN2*, and *POSTN*. KEGG pathway analysis also identified downregulation in important pathways of glutathione metabolism, DNA replication, cell signaling, and biosynthesis of amino acids. These data suggest a pivotal role for CEBPD in regulating MC activation and fibrotic differentiation.

### CEBPD is a key regulator of fibrotic function in CLAD MCs.

To confirm that increased *CEBPD* expression is associated with CLAD disease state, CEBPD was evaluated at the protein level in a separate cohort of CLAD and non-CLAD MCs matched by time after transplantation (Supplemental Figure 7A). Matched pairs were obtained from patients within 4 months (±1 day) of each other (Supplemental Table 2). Significantly higher CEBPD protein expression was noted in CLAD MCs (paired *t* test, *P* = 7.454 × 10^–5^), and linear regression analysis demonstrated no significant correlation between time after transplantation and CEBPD protein (non-CLAD: *r*^2^ = 0.004015, *P* = 0.8713; CLAD: *r*^2^ = 0.07175, *P* = 0.4859). To exclude any potential impact of culture conditions on CEBPD expression, MCs from patients with and without CLAD were cultured for 3 passages, and protein samples were collected and analyzed for the expression of CEBPD. Two-way repeated-measures ANOVA demonstrated a significant effect of group, with higher CEBPD protein expression in CLAD MCs (*P* = 0.0033), but no effect of passage (*P* = 0.4893) and no group × passage interaction (*P* = 0.4413), indicating that CEBPD expression was not altered by serial passaging (Supplemental Figure 7B).

**Figure 7:**
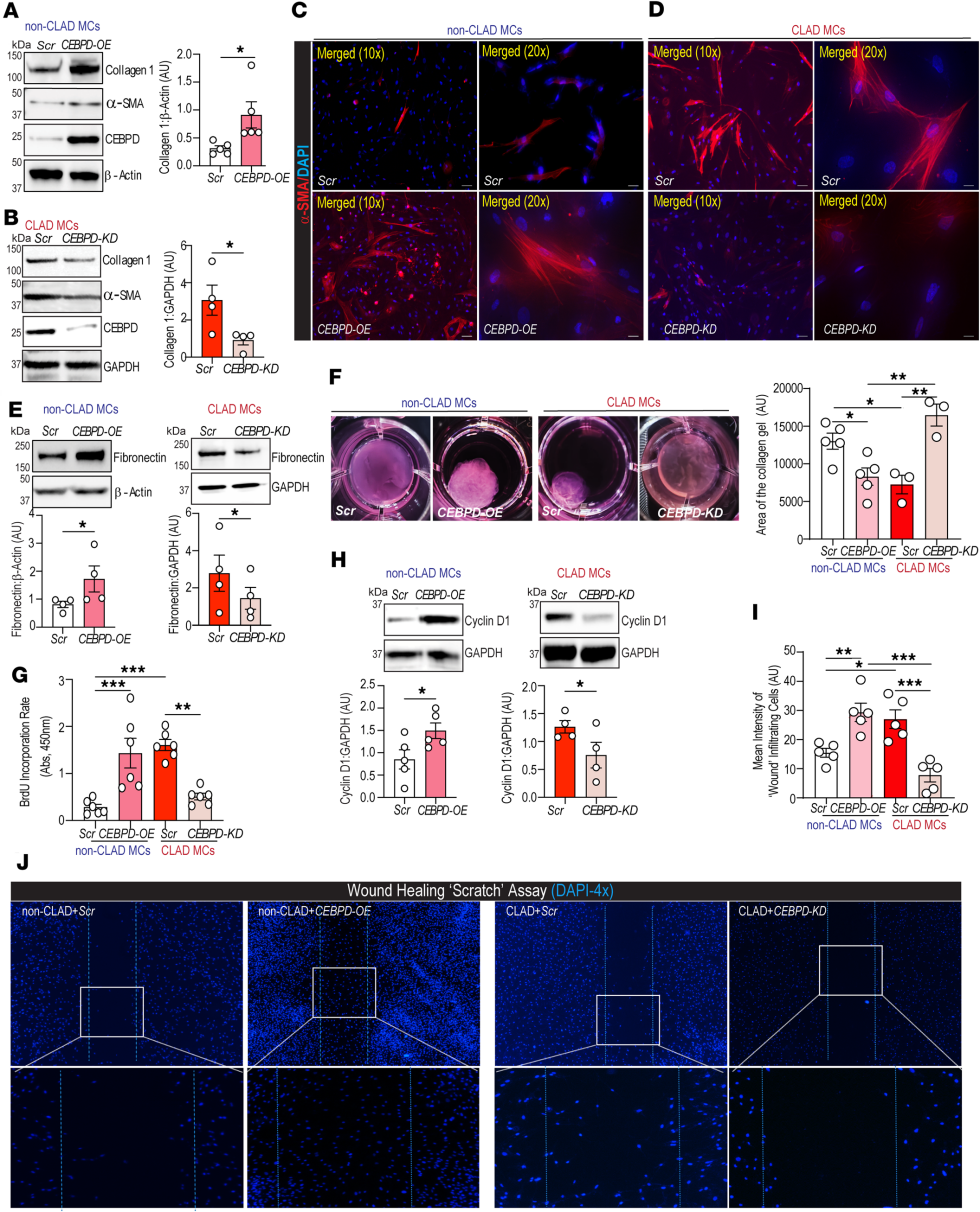
CLAD MC–associated CEBPD promotes fibrogenic functional competence, including mesenchymal contractility, matrix synthesis, proliferation, and migration. (**A** and **B**) Western blot analysis of collagen 1 with corresponding densitometry, together with α-SMA and *CEBPD* expression in *CEBPD*-overexpressing (*CEBPD-OE*) (**A**) and *CEBPD*-silenced (*CEBPD-KD*) (**B**) MCs. (**C** and **D**) Representative micrographs showing extent of differentiation via α-SMA expression in *CEBPD-OE* non-CLAD MCs (**C**) and loss of α-SMA signal in *CEBPD-KD* CLAD MCs (**D**). Nuclei were counterstained with DAPI. Original magnification, ×100 and ×200. Scale bars: 100 μm. (**E**) Western blot analysis of fibronectin with corresponding densitometry. (**F**) Contractile capacity assessed using a 3D collagen gel contractility assay, with quantification of gel area at 72 hours and representative gel images. (**G**) Proliferative potential assessed by BrdU incorporation following 6-hour labeling in *CEBPD*-*OE* non-CLAD MCs and *CEBPD*-*KD* CLAD MCs. (**H**) Western blot analysis of cell cycle progression marker, cyclin D1, with corresponding densitometry. (**I** and **J**) Migratory potential accessed by wound-healing “scratch” assay in MCs with *CEBPD*-*OE* or *CEBPD*-*KD*. Quiescent MCs that were wounded at baseline and analyzed after 72 hours for DAPI-stained cells infiltrating the wounded area (**I**). Images were captured at ×40 magnification showing the margins of the wound within dotted lines and cells infiltrating the wounded area. Inset: magnified ×40 images of cells in the wounded area. (**J**). Data in **A**, **B**, and **E**–**I** are represented as mean ± SEM. *P < 0.05, **P < 0.01; ***P < 0.001 by unpaired, 2-tailed Student’s *t* test (**A**, **B**, **E**, and **H**) or 1-way ANOVA with Bonferroni’s post hoc test (**F**, **G**, and **I**).

To further investigate the functional role of CEBPD in regulating fibrotic transformation of MCs in CLAD, lentiviral constructs were utilized to overexpress *CEBPD* in non-CLAD MCs (*CEBPD-OE*) and to silence *CEBPD* expression in CLAD MCs (*CEBPD-KD*). CEBPD was noted to regulate both ECM deposition and contractility, with *CEBPD* overexpression in non-CLAD MCs significantly increasing collagen I and α-smooth muscle actin (α-SMA) expression, whereas *CEBPD* silencing in CLAD MCs attenuated their constitutive expression (Figure 7, A and B). Immunofluorescence analysis confirmed enhanced α-SMA organization into contractile stress fiber networks in *CEBPD*-*OE* non-CLAD MCs, comparable to that observed in CLAD MCs, while *CEBPD* knockdown disrupted this cytoskeletal architecture (Figure 7, C and D). Consistent with the established role of CEBPD in fibronectin transcription (41), *CEBPD* overexpression induced robust fibronectin expression in non-CLAD MCs, whereas fibronectin levels were markedly reduced following *CEBPD* silencing in CLAD MCs (Figure 7E). Functionally, CEBPD-dependent cytoskeletal remodeling was associated with enhanced contractility, as measured by 3D collagen gel contraction. Gel contraction in *CEBPD*-*OE* non-CLAD MCs was comparable to CLAD controls but was significantly impaired in CLAD MCs following *CEBPD* knockdown (Figure 7F). CEBPD also regulated mesenchymal proliferative capacity. BrdU incorporation demonstrated increased proliferation in *CEBPD*-*OE* non-CLAD MCs and reduced proliferation following *CEBPD* silencing in CLAD MCs (Figure 7G). These changes were accompanied by corresponding alterations in cyclin D1 expression (Figure 7H). Finally, scratch assays revealed enhanced wound closure in *CEBPD-OE* non-CLAD MCs, whereas *CEBPD* silencing significantly impaired wound closure in CLAD MCs (Figure 7, I and J). Together, these data identify CEBPD as a key regulator of mesenchymal fibrogenic functions, including matrix production, contractility, proliferation, and motility.

### Cultured MCs retain and reflect their in vivo transcriptomic signatures.

**Figure 8:**
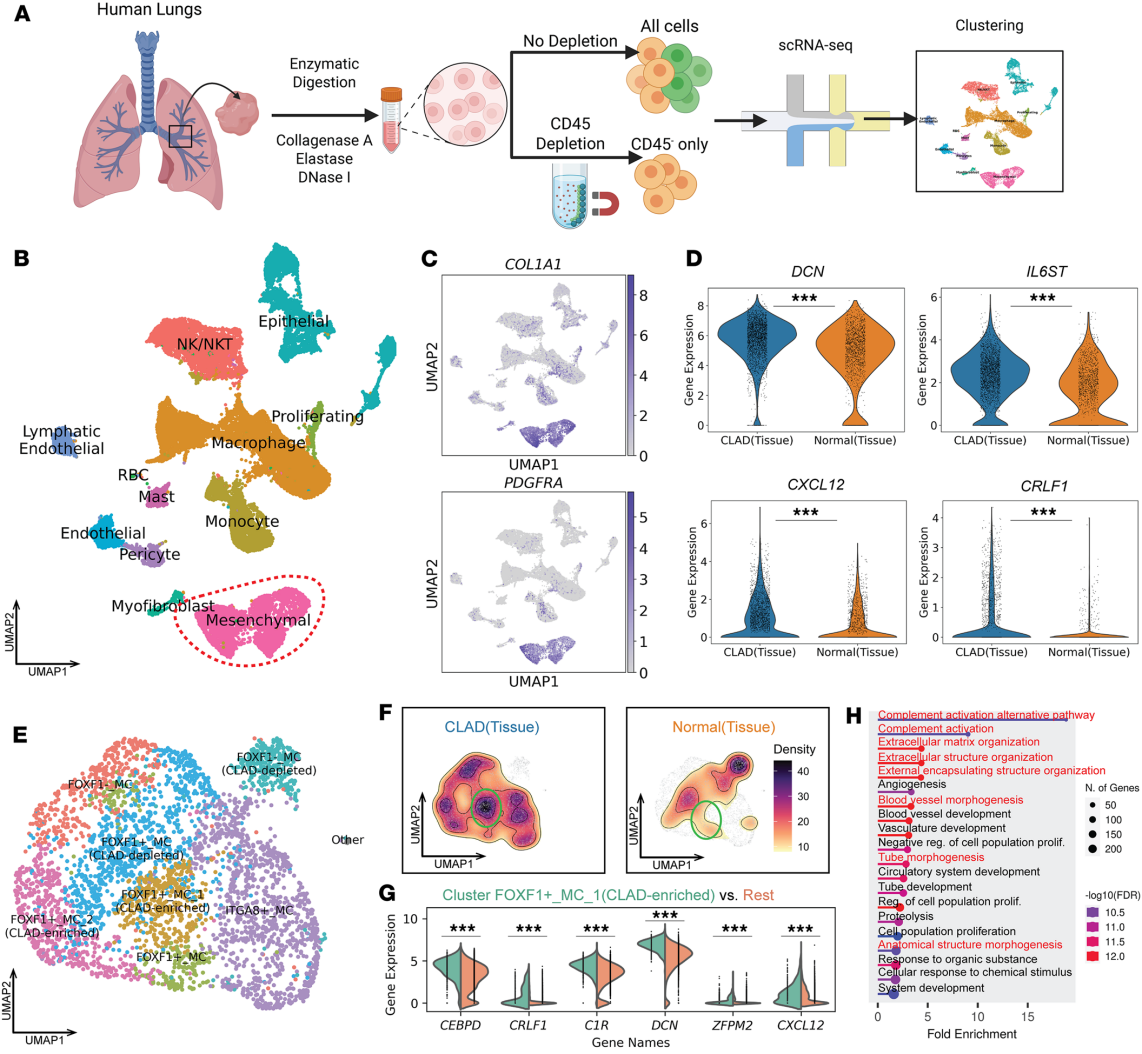
Cultured MCs retain and reflect their in vivo phenotype. (**A**) Overview of scRNA-seq sample preparation and analysis. Workflow was created using BioRender.com. (**B**) UMAP representation of 28,279 cells from both non-depleted and CD45^+^ cell–depleted samples. Each dot represents a single cell, and cells are colored by cell type. MCs are circled in a red dashed line. (**C**) UMAP colored by *COL1A1* (upper) and *PDGFRA* (bottom) expression. (**D**) Violin plots depicting the expression levels of *DCN* (upper left), *IL6ST* (upper right), *CXCL12* (bottom left), and *CRLF1* (bottom right) across conditions. The width of each plot represents the distribution of expression values. Comparisons are shown between CLAD tissue (blue) and normal tissue (orange). (**E**) UMAP representation of 3,902 tissue MCs. (**F**) Density plots of integrated tissue MCs (left: CLAD tissue; right: normal tissue). (**G**) Split violin plots showing the expression levels of *CEBPD*, *CRLF1*, *C1R*, *DCN*, *ZFPM2*, *CXCL12*, and *NFIB*. Each violin is split to compare cluster FOXF1^+^_MC_1 (CLAD-enriched) with the rest of the MCs, with the width representing the distribution of expression values. (**H**) GO enrichment analysis showing upregulation of complement activation–related and ECM-related genes in CLAD tissue MCs. ***P ≤ 0.001.

To determine whether our cultured MCs retain and reflect their in vivo phenotype, we analyzed MCs directly isolated from CLAD human lung autopsy samples. We collected and performed scRNA-seq on cells from human lung autopsy samples, including 1 from a CLAD patient and 2 from healthy controls. Due to the low number of MCs detected in these initial samples (Supplemental Figure 8A), we sequenced 2 additional samples (1 from the same CLAD patient and 1 from the same healthy control) after CD45^+^ cell depletion (Figure 8A).

After quality control filtering, we obtained 28,279 cells from both non-depleted and CD45^+^ cell–depleted samples. We performed integration and unsupervised clustering using LIGER (30, 31), and identified non-immune cell types including mesenchymal, endothelial, epithelial cells, pericytes, and myofibroblasts as well as immune cell types including macrophages, monocytes, mast cells, natural killer (NK)/natural killer T (NKT) cells, red blood cells (RBCs), and proliferating cells (Figure 8B). A total of 4,254 tissue MCs were identified based on the expression of *COL1A1* and *PDGFRA* (Figure 8C). Differential expression analysis between CLAD tissue MCs and healthy controls revealed 5,263 upregulated genes and 5,130 downregulated genes in CLAD tissue MCs. To assess the transcriptional similarity between CLAD tissue MCs and CLAD cultured MCs, we compared these DEGs with the previously identified CLAD signature in Figure 2 from the logistic regression model. Notably, 9 out of 17 upregulated genes in CLAD cultured MCs were also significantly upregulated in the CLAD tissue MCs (Figure 8D and Supplemental Figure 8B). Of these, 5 genes (*DCN*, *IL6ST*, *CXCL12*, *CRLF1*, and *NTNG1*) were predicted as the downstream targets of CEBPD based on the eGRN, and 4 genes (*IL6ST*, *CXCL12*, *NTNG1*, and *NFIB*) had previously been shown to be downregulated after *CEBPD* silencing. Additionally, 6 out of 30 downregulated genes in CLAD cultured MCs were also significantly downregulated in the CLAD tissue MCs, with 1 gene (*ID1*) previously shown to be downregulated after *CEBPD* silencing (Supplemental Figure 8C). These results suggest that MCs in vivo shared key transcriptomic signatures with their cultured counterparts.

To obtain a more fine-grained resolution of cell states in tissue MCs, we performed subclustering (Figure 8E). This analysis identified a subcluster, FOXF1^+^_MC_1 (CLAD-enriched), which was specifically enriched in CLAD samples (Figure 8F and Supplemental Figure 9A). Importantly, this subcluster exhibited the highest expression of *CEBPD* compared with other clusters, consistent with the fibrotic state observed in vitro (Figure 8G). Additionally, 5 genes (*CRLF1*, *C1R*, *DCN*, *ZFPM2*, and *CXCL12*), predicted as downstream targets of CEBPD based on the eGRN, were significantly upregulated in this subcluster (Figure 8G). Furthermore, 2 CLAD-upregulated signature genes (*NFIB* and *COL3A1*) were also significantly upregulated (Supplemental Figure 9B). To further assess the enrichment of our signature genes, we performed gene set enrichment analysis (GSEA) (42, 43). The analysis revealed significant enrichment in the upregulated signature, with a normalized enrichment score (NES) of 1.97. Although the whole downregulated gene set was not significantly enriched, some of the genes still were significantly downregulated in this subcluster (Supplemental Figure 9C). GO enrichment analysis of the DEGs in this subcluster revealed significant upregulation of biological processes related to ECM organization, ECM structural organization, and external encapsulating structure organization, suggesting their fibrotic phenotype and aligning with the top downregulated GO terms observed after *CEBPD* silencing (Figure 8H). Additionally, several GO terms associated with morphogenesis were enriched, consistent with those observed in the fibrotic state of MCs in vitro. These results suggest this subcluster may represent the in vivo counterpart of the fibrotic state identified in vitro.

## Discussion

Fibrosis is an important pathogenic finding in chronic allograft failure across all solid organ transplants. However, targeting fibrogenesis continues to be elusive, with fibrotic MCs demonstrating multiple dysregulated pathways. In this study, we leveraged genetic multiplexing and single-cell multiomics to provide an unbiased examination of distinct MC gene expression and chromatin accessibility signatures in lung allografts undergoing early inflammatory and late fibrotic processes. Genetic multiplexing — pooling cells from multiple donors and demultiplexing them based on natural genetic variation — enabled us to reduce batch effects and increase the number of cells and donors profiled in our study. These combined transcriptomic and epigenomic investigation identified a unique gene signature in CLAD patient–derived MCs and nominated CEBPD as a candidate TF driving gene regulatory alterations in CLAD MCs. A shift in composition toward a fibrotic state was uniquely identified in MCs derived from patients after CLAD onset but not noted in MCs from CLAD-free patients or MCs isolated early after transplantation in patients who later developed CLAD. Knocking down *CEBPD* with siRNA in CLAD MCs partially reverted the CLAD transcriptomic signature and identified downstream targets like immunomodulatory cytokine *CXCL12* and signal-transducing subunit *IL6ST*. Furthermore, a functional role for CEBPD as a regulator of fibrotic differentiation of MCs in CLAD was confirmed using overexpression and silencing approaches in non-CLAD and CLAD cells, where CEBPD was shown to regulate ECM deposition, contractility, proliferation, and migration. Together, these data provide insight into an MC transcriptional state shift that accompanies CLAD and position CEBPD as an important regulator of MC fibrotic functions with potential implications for tissue fibrosis and chronic allograft dysfunction.

Our unbiased analysis of the global transcriptome of MCs isolated from transplanted lungs reveals a distinct MC transcriptional signature in patients with CLAD, with CLAD-derived MCs exhibiting a pronounced shift toward a fibrotic state. CLAD MCs could be differentiated from non-CLAD with 99.2% accuracy and specific upregulated and downregulated genes were identified. An important control was our comparison of MCs isolated from patients early after transplantation, where differentiation between the 2 groups was not evident. CLAD emerges over time, with the incidence of CLAD increasing over time after transplantation. The early-posttransplant group included patients who developed CLAD later in the course of their transplant, with an average time to CLAD of 3.5 years (10). MCs isolated at the early stage could not be classified with high accuracy into diseased and non-diseased groups, and these cells were compositionally similar to each other and the non-CLAD later-posttransplant control group. Investigation of compositional heterogeneity using unsupervised clustering also provided insight into the emergence of a fibrotic state with enrichment of this cluster in CLAD MCs. This cluster demonstrated upregulation of biological processes like positive regulation of transcription by RNA polymerase II, cell surface receptor signaling pathway, regulation of cell differentiation, cell communication, signal transduction, and cell motility. In MCs derived early after lung transplantation, we observed a predominance of an inflammatory cluster characterized by upregulation of neutrophil activation. However, there were no differences in the inflammatory cluster proportion between patients who later developed CLAD or remained CLAD-free. While more work needs to be done to understand this inflammatory phenotype of MCs and how it affects the local immune milieu as well as the later fibrotic transformation, at this time, our data do not suggest an inherent predisposition or an early MC skewing in CLAD. Instead, our findings provide evidence for an acquired, stable fibrogenic transformation in CLAD MCs, offering further insight into our understanding of immune injury–associated fibrotic processes, where immune regulation after disease onset fails to slow disease progression.

A key finding was the identification of the TF CEBPD as a key regulator of the fibrotic state of MCs in CLAD. CEBP family members, specifically CEBPD, were dominant TFs identified in analyses of chromatin accessibility profiles of CLAD MCs and their binding motifs using motif scanning. Its specificity to the CLAD state was suggested by a parallel analysis of early-posttransplant MCs, where CEBPD was not noted among their enriched motifs. Gene regulatory analysis combining single-cell transcriptomic and epigenomic features nominated CEBPD as a key regulator of the CLAD-enriched fibrotic state, with both the most state-specific expression in the fibrotic state and the most predicted targets. The bulk RNA-seq data demonstrated that silencing *CEBPD* downregulated key fibrotic genes and shifted the overall gene expression profile of CLAD MCs toward a less fibrotic state. Our investigation of time after transplantation matched MCs from CLAD and non-CLAD patients confirmed constitutively stable CEBPD protein expression in CLAD MCs. Importantly, inhibiting *CEBPD* expression in CLAD MCs reversed their fibrotic phenotype, whereas its endogenous overexpression in non-CLAD MCs imparted them with a proliferative, migratory, and contractile phenotype similar to the CLAD state. While very few studies have investigated CEBPD in MCs in the context of fibrosis, CEBPD has been demonstrated to be the master TF regulating ECM proteins, including fibronectin, and several collagens in glioblastoma (41). Our findings were similar, with a decrease in collagen I and fibronectin noted in CLAD MCs with *CEBPD* silencing and an increase in these proteins noted in non-CLAD cells overexpressing *CEBPD*. ECM structural constituent was also the most markedly downregulated GO term in our analysis of *CEBPD*-*KD* CLAD MCs. Among the downregulated ECM genes were periostin (*POSTN*) and thrombospondin1 (*TSP1*), both key players in fibrosis along with fibrillin 1 and 2 (*FBN1/2*), and multiple collagens. Positive regulation by CEBPD on α-SMA protein expression, the primary marker of myofibroblasts along with cellular contractility as shown by gel contraction assay, further implicates it in MC activation and differentiation. Importantly, higher baseline α-SMA expression and contractile phenotype of CLAD MCs were reversed by *CEBPD* silencing, suggesting reprogramming and dedifferentiation with CEBPD targeting. While CEBPD has not been studied in lung fibrosis or in primary adult lung MCs, an investigation of embryonic lung fibroblasts in the context of chemotherapy has previously demonstrated that endogenous induction of CEBPD upregulates α-SMA and TGF-β1 transcripts and the loss of CEBPD attenuates CDDP- and 5-FU–induced transcription of α-SMA and TGF-β1 genes (44). The enhanced proliferative and migratory phenotype imparted by CEBPD expression in non-CLAD MCs and abrogation of proliferation and migration in *CEBPD-KD* CLAD MCs further suggested that CEBPD is not merely associated with the fibrotic cellular state but plays a direct and functional role in regulating MC activation and fibrotic differentiation. Future studies are needed to investigate the cell-specific role of CEBPD in fibrosis and to validate a requisite role for CEBPD in CLAD pathogenesis using in vivo models. However, regulation of a master TF to reverse the fibrogenic state of MCs represents a promising therapeutic target for mitigating fibrosis, and our investigations suggest CEBPD to be one such putative regulator.

Among the genes identified in this unbiased approach are targets that are being increasingly recognized across other fibrotic diseases and have a direct link to prior investigation of the mechanisms of lung allograft fibrosis. *CXCL12*, *IL6ST*, and *CRLF1* were found to be (a) upregulated in CLAD MCs in an unbiased genomic analysis; (b) were confirmed to be downregulated regulated by *CEBPD* silencing by RT-qPCR, and importantly; (c) shown to be significantly upregulated in fibrotic MCs in single-cell examination of CLAD lung tissue. Interestingly, all 3 of these pathways link to our previously described signaling pathways in CLAD MCs. We have recently characterized *CXCL12* expression by *Foxf1*^+^ MCs and its role as a regulator of graft ASC niche in RAS (45). IL6ST (also known as glycoprotein 130 [GP130]), the universal signal-transducing β-receptor subunit for all IL-6–related cytokines, allows for IL-6 trans-signaling in cells lacking the IL-6 receptor (IL-6R) (46), and we have demonstrated that IL-6 trans-signaling regulates both MC fibrotic differentiation (17) and *CXCL12* expression (45). Interestingly, others have shown that GP130 upregulation can create an autocrine activation loop involving IL-6/soluble IL-6R (47). Cytokine receptor–like factor 1 (CRLF1) has been implicated in fibrosis in a variety of tissues (48–50), with specific relevance to our previous findings of increased mTORC2 signaling (51) and upregulation of critical mTORC2 component Sin1 in CLAD MCs (16). CRLF1 has been identified as an mTORC2 component that strengthens the interaction between Sin1 and AKT, enhancing AKT Ser473 phosphorylation (52). *CXCL12* and *IL6ST* were also found to be direct downstream targets of CEBPD, further linking CEBPD to the fibrotic cell state.

The diagnosis of CLAD is made clinically using spirometric decline, as transbronchial biopsies are not sensitive enough to offer histologic confirmation. CLAD is classified into clinical phenotypes based on spirometric decline pattern and radiographic features (10). While some patients are classified into the 2 predominant clinical phenotypes of bronchiolitis obliterans syndrome and restrictive allograft syndrome, others fall into the indeterminate or mixed category. Fibrosis of the allograft is common to all phenotypes, with fibrosis emanating from bronchovascular bundles being an important central feature in the majority of rejecting grafts. Our previous work on MCs in BAL has offered a window into fibrogenesis in a transplanted lung. We have shown that increased numbers of MCs in BAL, as quantified by colony-forming unit (CFU) assay, precede the development of CLAD, with MC CFUs noted to be a strong predictor of CLAD onset (14). Furthermore, high MC CFUs in BAL at CLAD onset were associated with poor survival and restrictive allograft syndrome diagnosis (15). The CLAD MCs utilized in this multiomic study were from patients with diverse clinical CLAD phenotypes, and the present study does not have the power to address the specific correlation with CLAD phenotypes. Further work is needed to understand whether epigenetic changes in CEBPD mark a specific clinical or molecular phenotype and will require a large study sample with outcome analysis. Our study included MCs from lungs after CLAD onset or very early after transplantation. While this allowed us to determine whether CLAD-associated changes are acquired over time, a longitudinal study of MCs at various time points in patients with variable posttransplant complications, like acute rejection or infection episodes, can shed more light on the time course of emergence of this fibrogenic phenotype and the preceding insults.

In conclusion, our single-cell multiomics analysis revealed key molecular signatures and regulatory networks associated with fibrosis and CLAD. The identification of a distinct transcriptomic signature, and a skewing toward a more homogeneous fibrotic state in CLAD MCs underscores the acquisition of a stable fibrogenic transformation in graft MCs that drives CLAD. What we believe to be the first identification of the TF CEBPD as a master regulator of the fibrotic cell state and links it to key downstream genes in MCs. Future studies should focus on further dissecting the regulatory mechanisms at play and exploring combinatorial therapeutic strategies to effectively combat fibrosis and chronic rejection in lung transplantation.

## Methods

See Supplemental Methods for additional methodology.

### Sex as a biological variable.

MCs were cultured from BAL fluid samples derived from lung transplant patients of both female and male sex (Supplemental Table 1). None of the experiments or analyses were limited to samples from either sex. As such, sex was not evaluated as a biological variable in the experiments.

### MC isolation from human lung allografts and cell culture.

BAL samples were obtained from human lung transplant recipients under a protocol for human studies approved by the University of Michigan IRB. MCs were isolated from BAL as previously described (6, 7, 11, 13, 14, 16–18). Briefly, all cells isolated from BAL fluid were pelleted by centrifugation (400*g* for 5 minutes) and were maintained in adherent culture conditions. MCs were identified by their growth as CFUs with fibroblastoid morphology. Cells were maintained in culture in high-glucose 1× DMEM (Gibco, Thermo Fisher Scientific) supplemented with 10% FBS (Avantor), 100 U/mL penicillin/streptomycin (Gibco), and 0.5% Amphotericin B (Gibco), at 37°C in humidified incubators with 5% CO_2_ for no less than 1 week and no more than 3 weeks following initial sample processing before being passaged by trypsinization. Individual BAL samples and subsequent MCs isolated were treated as separate cell lines. Cells from passages three through 6 were used for all experiments. No significant difference between passages was observed (Supplemental Figure 7A). CLAD was defined by a greater than 3-week persistent decline in forced expiratory volume in 1 second (FEV1) and forced vital capacity (FVC) utilizing ISHLT guidelines and as previously described (10, 53, 54). CLAD phenotyping was performed using the latest ISHLT guidelines (10). At the early time point (<6 weeks after transplantation), samples were collected from patients who had not yet developed CLAD but were later clinically classified as having developed CLAD (*n* = 4) or remained CLAD-free (*n* = 3). At the late time point (>1 year after transplantation, samples were obtained from patients with CLAD (*n* = 3) and without CLAD (*n* = 2) (Supplemental Table 1). We previously demonstrated that these MCs are donor-derived rather than recipient-derived (11), which was further validated here by checking the sex chromosome gene expression (Supplemental Figure 10).

### Cell preparation for single-cell multiome sequencing.

MCs were plated on 10 cm dishes and grown to full confluence. For 10X multiome RNA-seq and ATAC-seq, nuclear isolation was performed according to demonstrated protocols from 10X Genomics (CG000366, Revision C). Briefly, culture media were aspirated, and plates were washed twice with cold PBS. Cells were isolated using a cell scraper and 5 mL of ice-cold PBS into a 15 mL conical tube and spun at 500*g* at 4ºC. The supernatant was removed and the cells were resuspended using 1× lysis buffer containing 10 mM Tris-HCl (Invitrogen), 10 mM NaCl (Thermo Fisher Scientific), 3 mM MgCl_2_ (Promega), 0.1% Tween 20 (Thermo Fisher Scientific), 0.1% Nonidet P40 Substitute (Sigma-Aldrich), 0.01% digitonin (Thermo Fisher Scientific), 1% BSA (Sigma-Aldrich), 1 mM DTT (Sigma-Aldrich), and 1U/μL RNasin Plus Ribonuclease Inhibitor (Promega) in Nuclease-Free Water (Invitrogen) and incubated on ice for 5 minutes. Cells were monitored until less than 10% of cells remained unlysed. The pellet was then washed 2 times with centrifugation at 500*g* for 5 minutes at 4°C, each time removing the supernatant. Finally, 10,000 target nuclei were resuspended in a diluted nuclei resuspension buffer (10X Genomics), pooling 2–3 cell lines for each submission.

### Single-cell multiome library generation.

Single-cell multiome libraries were prepared using the Chromium Next GEM Single Cell Multiome ATAC and Gene Expression kit (10X Genomics) according to the manufacturer’s instructions. ATAC-seq, library preparation, and 3′ gene expression was performed with 50,000 reads per cell depth. Sequencing was performed by the Advanced Genomics Core at the University of Michigan using a NovaSeq 6000 (Illumina) platform.

### Logistic regression classifier training.

The scRNA-seq gene expression matrix was utilized and trained logistic regression classifiers for CLAD versus non-CLAD. We used logistic regression with an elastic-net penalty, a popular approach for extracting a small set of features that can generalize well despite a relatively small number of samples. Elastic-net regression uses a linear combination of the L1 penalty (lasso) and the L2 penalty (ridge), leading to sparse selection of a subset of features without arbitrarily choosing among correlated predictors (55–57). Scikit-learn (version 1.2.2) (58) was used to perform the preprocessing and training. To build our signature from genes with appreciable expression levels, we filtered for genes with greater than 5,000 unique molecular identifiers (UMIs) across all cells. Additionally, to prioritize genes with specific expression in mesenchymal cells, we filtered out genes with a total number of UMI counts exceeding 30,000 in non-mesenchymal cell types. Genes encoded on the mitochondrial chromosome, ribosomal protein genes, and genes encoded on sex chromosomes were additionally removed. A total of 5,022 genes for CLAD versus non-CLAD genes remained after filtering and were used to train the logistic regression model. Next, counts were normalized by the total expression per cell using the *normalize* function. The normalized counts were then log-transformed. Given that single-cell sequencing generated different numbers of cells per condition resulting in a skewed class distribution, upsampling was applied to create balanced class distributions. The minority class was oversampled to match the number of cells in the majority class using the RandomOverSampler function from the imbalanced-learn package (version 0.10.1) (59). The preprocessed data were partitioned into training and testing sets in a ratio of 9:1. Testing data were used to evaluate the model performance and were held out from training. Before training the model, each gene feature in the training dataset was normalized to zero for mean and unit variance using the StandardScaler function. A logistic regression model with Elastic-Net penalty was then trained using the SGDClassifier (loss=’log’, penalty=’elasticnet’, l1_ratio=0.5). Five-fold cross-validation was performed to select the α and evaluate the model performance using the GridSearchCV function. To further validate gene signatures from our classifier, we performed RT-qPCR on a selected set of genes.

### scATAC-seq data analysis.

ArchR (version 1.0.1) (29) was used for the intersample analysis of scATAC-seq data. Pseudo-bulk replicates were first generated to obtain measurements of statistical significance using the addGroupCoverages function with default parameters. Reproducible merged peak sets were generated using the addReproduciblePeakSet function, which first called peaks per dataset using MACS2 and then iteratively merged overlapping peaks across datasets. Marker peaks were identified using the getMarkerFeatures function and annotated using the peakAnnoEnrichment function to obtain enriched motifs. Pairwise Wilcoxon testing was performed between CLAD and non-CLAD groups and the cutoffs were an FDR of 0.05 or less and absolute log_2_(fold change) of 1 or greater.

### scRNA-seq data integration.

scRNA-seq data matrices after quality control were integrated using PyLiger (version 0.0.1) (31), a Python version of our previously developed algorithm, LIGER (30). LIGER is a widely used package for single-cell multiomic data integration. Briefly, it employs integrative non-negative matrix factorization (iNMF) to identify shared and dataset-specific metagenes of cellular variation. Before integration, scRNA-seq data were first analyzed with standard preprocessing steps required by LIGER including normalization, highly variable gene selection, and scaling to unit variance. Joint matrix factorization was performed on the normalized and scaled datasets followed by quantile normalization. We then clustered the cells using the Leiden algorithm. Given that we noticed biological variation among cells from different donors, we performed LIGER by donor; the count matrices were split into donor-specific count matrices based on donor identity assigned during genetic demultiplexing. The density plots were visualized using customized R code (https://welch-lab.github.io/liger/reference/plotDensityDimRed.html). The compositional changes were evaluated by calculating the proportion of cell types within each sample and visualized using the *boxplot* function from scCODA (60).

### GO enrichment analysis.

The Wilcoxon rank-sum test was conducted using the *runWilcoxon* from LIGER to identify DEGs. We filtered DEGs based on an adjusted *P* value of less than 0.05 and an absolute log_2_(fold change) greater than 2 to identify both upregulated and downregulated genes. GO enrichment analysis and network visualization were subsequently performed on the DEGs using ShinyGO (version 0.77) (61). Default parameter settings in ShinyGO were applied, including an FDR cutoff of 0.05, a minimum pathway size of 2, a maximum pathway size of 5,000, and a display of the top 20 enriched pathways.

### RNA velocity analysis.

Spliced and unspliced counts of each sample were generated using Velocyto (62). To correct the potential batch and donor differences and prepare input for VeloVAE, both RNA and ATAC matrices as well as spliced and unspliced counts were integrated using Seurat’s *FindIntegrationAnchors* and *IntegrateData* functions. The corrected matrices were used to train a VeloVAE model (35). The velocity stream plot was visualized using the *velocity_embedding_stream* function from scVelo.

### eGRN analysis.

SCENIC^+^ was employed to build an eGRN (36). The SCENIC^+^ workflow consists of 3 analysis steps: (a) using the pycisTopic module to identify candidate enhancers, (b) using the pycisTarget module to identify enriched TF-binding motifs, and (c) using the SCENIC^+^ module to link TFs to candidate enhancers and target genes.

### pycisTopic.

The consensus peak set generated by ArchR was used to identify candidate enhancers. Topic modeling was performed using the *run_cgs_models* function from pycisTopic, with the number of topics tested set to 5, 10, and 15.

### Motif enrichment analysis.

In the second step, the *run_pycistarget* function from pycisTarget was used to perform motif enrichment analysis with default parameters. The precomputed cisTarget database was downloaded from the pycisTarget website (https://resources.aertslab.org/cistarget/databases/homo_sapiens/hg38/screen/mc_v10_clust/region_based/), accessed December 22, 2022.

### Inferring eGRNs using SCENIC^+^.

Finally, the *run_scenicplus* function from the SCENIC^+^ module was used to construct eGRNs. We used default parameters. The heatmap/dotplot was generated using the *heatmap_dotplot* function on significant eRegulons. TF cooperation activities were calculated using the *jaccard_heatmap* function. The network was visualized using the *plot_networkx* function.

### Bulk RNA-seq.

CLAD MCs treated with either scrambled (non-targeting) control siRNA or CEBPD siRNA were harvested 24 hours after transfection. Total RNA was isolated using the RNeasy Mini Plus Kit (Qiagen) according to the manufacturer’s instructions. RNA was quantified via measuring absorbance at 260/280 nm using a NanoDrop 2000 (Thermo Fisher Scientific) prior to submission to the Emory Integrated Genomics Core (EIGC). After RNA quality assessment was performed using a TapeStation system (Agilent Technologies), all mRNA was sent to Novogene for sequencing. Bulk RNA-seq (eukaryotic RNA-Seq, non-directional, stranded library) was conducted by Novogene using the Illumina NovaSeq PE150 platform that employs a paired-end sequencing technology yielding 150-bp read lengths, with all raw reads first cleared of adapter sequences.

### Bulk RNA-seq data alignment and differential expression analysis.

Raw RNA-seq reads from both treated and control groups were aligned to the GRCh38 reference genome using STAR (v.2.7.11a) (63) with default parameters. STAR produced sorted BAM files, which were used as the input for HTSeq-count (v.2.0.8 (64) to generate gene-level read counts. The raw gene counts were then imported into DESeq2 (v.1.40.2) (65) for differential expression analysis. DESeq2 was used to measure the effect of the condition (scrambled siRNA vs. CEBPD siRNA), controlling for donor differences. We used DESeq2 to normalize the data to account for differences in sequencing depth and other technical variations, using the median of ratios method. Statistical analysis was performed using the Wald test, and differentially expressed genes were identified using an adjusted *P*-value threshold of less than 0.05. Note that we did not set a cutoff for log_2_(fold change) to keep genes with small but statistically significant expression changes, ensuring that statistically significant but subtle alterations in gene expression were not excluded from downstream analyses. GO enrichment analysis and network visualization were subsequently performed on the DEGs using ShinyGO (version 0.80). Default parameter settings in ShinyGO were applied, including an FDR cutoff of 0.05, a minimum pathway size of 2, a maximum pathway size of 5,000, and a display of the top 20 enriched pathways.

### CLAD and normal human lung samples.

Normal human lungs were procured via Gift of Life (Michigan) and with the help of Steven K. Huang (University of Michigan Medical School). CLAD explanted lungs were procured at the time of retransplantation under an IRB-approved protocol. Representative tissue sections from small airway regions were excised using dissection scissors, manually minced, then placed into a digestion solution containing 1 mg/mL collagenase A (Sigma-Aldrich), 1 U/mg/mL elastase (Worthington), and 0.1 mg/mL DNase I (Thermo Fisher Scientific) in serum-free 1× high-glucose DMEM. Solutions were incubated for 45 minutes to 1 hour at 37°C in a dry incubator on an end-to-end rotator, mechanically disrupted, and incubated a second time for another 15–30 minutes. Cell suspensions were then filtered through 100 μm strainers and pelleted by centrifugation at 400*g* for 10 minutes. After aspiration of digestion solution and resuspension, cells were counted and either frozen or taken for CD45^+^ cell depletion.

### CD45^+^ cell depletion.

For CD45^+^ cell depletion, 8–10 million frozen cells per tube were segregated from lung digest solutions and placed on ice before being resuspended at a concentration of 2 × 10^6^ cells/0.5 mL in 1× serum-free DMEM (Gibco). Cells were then incubated with 1 μL of anti-CD45-Biotin antibody (Thermo Fisher Scientific, 13-0451-82) per 2 × 10^6^ cells at 4°C for 30 minutes on a rotator. Cells were then pelleted, washed twice, then incubated with High Capacity Magne Streptavidin Beads (Promega) for another 30 minutes at 4°C. Following magnetic capture, remaining CD45^–^ cells were counted, then frozen.

### Primary human lung digest cell submission for scRNA-seq.

After initial thawing of frozen cells, they were spun down and resuspended in PBS and kept on ice. A small aliquot of cells was taken at time of submission to monitor for cell death. Samples with greater than 70% viability were submitted for 3′ scRNA-seq as described above, without additional multiomic sequencing steps.

### Lentiviral transduction in lung-resident MCs.

Lentiviral particles driven by *EF1α* or *U6* promoters were generated using constructs for the overexpression of *CEBPD* (LV[Exp]-EGFP/Puro-EF1A>hCEBPD[NM_005195.4]; VectorBuilder, VB900179-5101njq) or silencing of *CEBPD* (pLV[shRNA]-EGFP/Puro-U6>hCEBPD[shRNA#1]; VectorBuilder, VB900036-5809dkr) or the respective scrambled controls (pLV[Exp]-EF1A>TagBFP2 and pLV[shRNA]-TagBFP2/Hygro-U6>Scramble_shRNA; VectorBuilder, VB010000-9640qzc and VB010000-0018fwc, respectively).

### Statistics.

Student’s 2-tailed *t* test was used to compare the means of 2 groups to determine significance. One-way ANOVA was used to compare the means of 3 or more groups, with a post hoc Bonferroni’s test to determine which groups showed significant differences unless otherwise specified. A *P* value of less than 0.05 was considered significant and was analyzed using GraphPad Prism (version 10.0.1) for 64-bit Windows.

### Study approval.

This study was performed in accordance with relevant guidelines and regulations described in a protocol for human studies approved by the University of Michigan IRB (approval number HUM00042443) in compliance with the Helsinki declaration, and all participants had provided written, informed consent prior to study participation.

### Data availability.

The raw and processed 10X multiome, bulk RNA-seq and 10X scRNA-seq data are available through the NCBI Gene Expression Omnibus (GSE281481, GSE281482, and GSE282248). Values for all data points shown in graphs are reported in the Supporting Data Values file.

## Author contributions

Conceptualization: VNL and JDW. Methodology: VNL, JDW, NW, PM, and LL. Experimentation: PM, NW, RV, SR, and FF. Analysis of single-cell and bulk RNA-seq data: LL. Resources: VNL and JDW. Writing, reviewing, and editing: LL, PM, RV, NW, SR, JDW, and VNL. All authors read and approved the final manuscript.

## Conflict of interest

The authors have declared that no conflict of interest exists.

## Funding support

This work was supported in part by NIH funding and is subject to the NIH Public Access Policy. Through acceptance of this federal funding, the NIH has been given a right to make the work publicly available in PubMed Central.

- Cystic Fibrosis Foundation grants LAMA21AB0 and LAMA16XX0 (to VNL and JDW).
- NIH/National Heart, Lung, and Blood Institute grants R01HL162171 and R01HL094622 (to VNL).

## Supplementary Material

Supplemental data

Unedited blot and gel images

Supporting data values

## Version 1 04/27/2026

In-Press Preview

## Version 2 07/08/2026

Electronic publication
