# Supplementary material for: Single-cell multiomic analysis of mesenchymal cells reveals molecular signatures and regulators of lung allograft fibrosis
Source: JCI Insight. 2026 Apr 23;11(13):e196651. doi: 10.1172/jci.insight.196651 (PMC13460852; doi:10.1172/jci.insight.196651)
Supplement: Supplementary file 1 [file jciinsight-11-196651-s116.pdf]

Single-cell multi-omic analysis of mesenchymal cells reveals molecular signatures and regulators of lung allograft fibrosis

Lu Lu[1], A. Patrick McLinden[2], Natalie M. Walker[3], Ragini Vittal[2], Yichen Wang[1], Fatima Fattahi[3], Stephen T. Russell[2], Michael P. Combs[3], Joshua D. Welch[1,4]*, Vibha N. Lama[2]*

SUPPLEMENTAL MATERIAL

1. Department of Computational Medicine and Bioinformatics, University of Michigan, Ann Arbor, MI, USA
2. Division of Pulmonary, Allergy, Critical Care, and Sleep Medicine, School of Medicine, Emory University, Atlanta, GA, USA
3. Division of Pulmonary & Critical Care, School of Medicine, University of Michigan, Ann Arbor, MI, USA
4. Department of Computer Science and Engineering, University of Michigan, Ann Arbor, MI, USA

* Co-Senior Authors
Please address correspondence to:
welchjd@umich.edu
vlama@emory.edu

Supplemental Methods

Genotyping and demultiplexing of single-cell multiome sequencing data. Cultured MCs from each cell line submitted for multiome sequencing were grown to confluence before their whole cell DNA was isolated using the DNeasy Blood and Tissue Kits (Qiagen, Beverly, MA) according to manufacturer's instructions. DNA concentrations were measured via absorbance at 260/280nm using a NanoDrop 2000 (ThermoFisher, Waltham, MA) prior to submission to the Advanced Genomics Core (AGC) at the University of Michigan. Demultiplexing of all 16 samples and associated pooled nuclei was conducted using an INF Multi-Ethnic Global-8 Kit (Illumina) genotyping kit. A set of SNPs was first obtained, then allelic expression of these variants in each cell was extracted using the read pileup approach (dsc-pileup from popscle for demuxlet or cellsnp-lite mode 1a for vireo). scRNA-seq and scATAC-seq were demultiplexed using demuxlet and Vireo separately. We observed doublet overestimation by both demultiplexing softwares (demuxlet and vireo; Supplemental Figure 5) in the RNA modality which was likely due to ambient RNA contamination. Therefore, the final assignment of cell identity was determined by taking into consideration both assignments from the RNA and ATAC modalities. Cells that have the same cell assignment between two modalities were kept. In addition, if a cell was identified as doublet in RNA, but not in ATAC, we used the cell assignment from its ATAC modality. We excluded cells that were ambiguous or identified as doublets in both modalities.

Single-cell multi-omic data preprocessing and quality control. Cell Ranger ARC pipeline (version 2.0.0) with default parameter settings was used to process the raw Chromium Single Cell Multiome ATAC + Gene Expression sequencing data. During alignment steps, reads were mapped to the human reference genome (GRCh38) provided by 10x Genomics. The filtered count matrices output by the pipeline were used for further analysis. Quality control steps were applied separately to the RNA and ATAC modalities, each using specific quality control metrics. For the RNA modality, low-quality cells with fewer than 200 unique genes or more than 20% mitochondrial

reads were removed. For the ATAC modality, we assess the data quality using four statistics: Fraction of Reads In Peaks (FRIP), Transcription Start Site (TSS) enrichment score, log number of unique fragments, and duplication rate (Supplemental Figure 4). Low-quality cells with fewer than 0.3 FRIP, 4 TSS enrichment score, or 3.3 log number of unique fragments were removed.

scRNA-seq data integration of human lung samples. The Cell Ranger ARC pipeline (verison 6.0.1 for non-delpelted samples and version 6.1.2 for depleted samples) with default parameter settings was used to process the raw Chromium Single Cell Gene Expression sequencing data. During alignment steps, reads were mapped to the human reference genome (GRCh38) provided by 10x Genomics. The filtered count matrices output by the pipeline were used for further analysis. Low-quality cells with fewer than 200 unique genes were first removed. scRNA-seq data matrices after quality control were integrated using PyLiger (version 0.0.1) (1). Before integration, scRNA-seq data were first processed with normalization, highly variable gene selection, and scaling to unit variance. Joint matrix factorization was performed on the normalized and scaled datasets followed by quantile normalization. We then clustered the cells using the Leiden algorithm. DEGs are identified using the Wilcoxon rank-sum test via the *runWilcoxon* function from LIGER (adjusted p-value threshold of < 0.05). Note that we didn't set a cutoff for log2FC.

Sub-clustering of tissue MC populations. MC populations identified from integration analysis were subset and re-clustering using LIGER (version 2.1.0). Factor loading of each sample was aligned using *centroidAlign* function. DEGs were calculated in the same way as in the scRNA-seq integration.

Gene ontology enrichment analysis. DEGs with adjusted p-value < 0.05 and a log fold-change > 1 was used for GO enrichment analysis and gene set enrichment analysis (GSEA). GO enrichment analysis was performed using ShinyGO (version 0.81). Default parameter settings in ShinyGO were applied. GSEA was performed using GSEAPY (version 1.1.3) with default parameters.

Western blot. *Protein isolation and immunoblotting.* MCs were plated and grown to 50% confluence in 60-mm dishes prior to serum starvation for 24 h. Total protein was collected from treated or untreated cells by whole cell lysate, lysed using Cell Lysis Buffer (10X, Cell Signaling, Danvers, MA) diluted to 1X with water and supplemented with 0.1M phenylmethylsulfonyl fluoride (PMSF) in ethanol (Sigma-Aldrich, Burlington, MA) and EDTA-free Halt Phosphatase Inhibitor Cocktail (Thermo Fisher Scientific, Waltham, MA). Whole cell lysates were then centrifuged (17,000 x g for 10 minutes) at 4°C. Lysate concentration was determined using a Pierce Coomassie Plus Assay Kit (Thermo Fisher Scientific) and analyzed via spectrophotometry (Thermo Scientific, BioMate 3 Spectrophotometer). Lysates were separated on 4–12% gradient Bis-Tris gels (Invitrogen) prior to immunoblot analysis. Sources of antibodies were as follows: Collagen I (catalog 72026S), Cyclin D1 (catalog 55506S), CEBPD (catalog 2318S), Fibronectin (catalog 26836S), GAPDH (catalog 97166), Cell Signaling; Beta-Actin (catalog A1978-100UL), α-smooth muscle actin (A5228-100UL), MilliporeSigma. HRP-conjugated anti-mouse and anti-rabbit secondary antibodies were A8924 (Sigma-Aldrich) and A0545 (SigmaAldrich), respectively. Immunoblots were imaged using a ChemiDoc MP (Bio-Rad). Densitometry analysis completed using ImageJ (NIH, Bethesda, MD).

CEBPD knockdown. *siRNA-mediated silencing transfection.* We transiently transfected CLAD MCs at 60% confluence with 100nM of ON-TARGETplus SMARTpool siRNA targeting either: *CEBPD* (Horizon) or a non-targeting control (Horizon). Briefly, cells were plated to 60% confluence in 60mm dishes before transient transfection in reduced serum containing media Opti-MEM (Gibco) with respective pools of siRNAs utilizing Oligofectamine (Invitrogen) for 24hrs. Following transfection, cells were maintained in serum-free medium for either 24 or 48 hrs prior to mRNA isolation or protein harvesting, respectively.

mRNA Isolation, cDNA Synthesis, and Gene Expression Analysis. Following siRNA-mediated transfection, cells were washed two times with PBS (Gibco) and lysed using RLT-buffer (Qiagen) supplemented with 2-Mercaptoethanol (Fisher Scientific). Total mRNA was isolated

immediately using RNeasy Plus Kit (Qiagen) according to the manufacturer's instructions. RNA quantity was assessed using a NanoDrop 2000 (Thermo Fisher). cDNA synthesis was performed using a High-Capacity cDNA Reverse Transcriptase Kit (Applied Biosystems) according to manufacturer's instructions. cDNA synthesis was conducted on a 96-well Veriti Thermocycler (Applied Biosystems). After cDNA synthesis, gene expression analysis with TaqMan gene expression master mix (Applied Biosystems) was performed with TaqMan assays (Applied Biosystems) for *ACTB* (Hs01060665_g1), *CXCL12* (Hs00171022_m1), *IL6ST* (Hs00174360_m1), *CRLF1* (Hs00191064_m1), *DCN* (Hs01081921_m1), *CEBPD* (Hs00270931_m1). All RT-qPCR assays were run on a QuantStudio3 Real Time PCR machine (Applied Biosystems).

Sequences used in lentiviral constructs. *hCEBPD* overexpression sequence:
*ATGAGCGCCGCGCTCTTCAGCCTGGACGGCCCGGCGCGCGGCGCGCCCTGGCCTGCGG
AGCCTGCGCCCTTCTACGAACCGGGCCGGGCGGGCAAGCCGGGCCGCGGGGCCGAGCC
AGGGGCCCTAGGCGAGCCAGGCGCCGCCGCCCCCGCCATGTACGACGACGAGAGCGCC
ATCGACTTCAGCGCCTACATCGACTCCATGGCCGCCGTGCCCACCCTGGAGCTGTGCCAC
GACGAGCTCTTCGCCGACCTCTTCAACAGCAATCACAAGGCGGGCGGCGCGGGGCCCCT
GGAGCTTCTTCCCGGCGGCCCCGCGCGCCCCTTGGGCCCGGGCCCTGCCGCTCCCCGC
CTGCTCAAGCGCGAGCCCGACTGGGGCGACGGCGACGCGCCCGGCTCGCTGTTGCCCG
CGCAGGTGGCCGCGTGCGCACAGACCGTGGTGAGCTTGGCGGCCGCAGGGCAGCCCAC
CCCGCCCACGTCGCCGGAGCCGCCGCGCAGCAGCCCCAGGCAGACCCCCGCGCCCGGC
CCCGCCCGGGAGAAGAGCGCCGGCAAGAGGGGCCCGGACCGCGGCAGCCCCGAGTACC
GGCAGCGGCGCGAGCGCAACAACATCGCCGTGCGCAAGAGCCGCGACAAGGCCAAGCG
GCGCAACCAGGAGATGCAGCAGAAGTTGGTGGAGCTGTCGGCTGAGAACGAGAAGCTGC
ACCAGCGCGTGGAGCAGCTCACGCGGGACCTGGCCGGCCTCCGGCAGTTCTTCAAGCAG
CTGCCCAGCCCGCCCTTCCTGCCGGCCGCCGGGACAGCAGACTGCCGGTAA; hCEBPD
shRNA* sequence: *TTGGGACATAGGAGCGCAAAGCTCGAGCTTTGCGCTCCTATGTCCCAA;*

Scrambled shRNA sequence:

CCTAAGGTTAAGTCGCCCTCGCTCGAGCGAGGGCGACTTAACCTTAGG

Scrambled BFP-Tag:

ATGAGCGAGCTGATTAAGGAGAACATGCACATGAAGCTGTACATGGAGGGCACCGTGGAC AACCATCACTTCAAGTGCACATCCGAGGGCGAAGGCAAGCCCTACGAGGGCACCCAGACC ATGAGAATCAAGGTGGTCGAGGGCGGCCCTCTCCCCTTCGCCTTCGACATCCTGGCTACT AGCTTCCTCTACGGCAGCAAGACCTTCATCAACCACACCCAGGGCATCCCCGACTTCTTCA AGCAGTCCTTCCCTGAGGGCTTCACATGGGAGAGAGTCACCACATACGAAGACGGGGGC GTGCTGACCGCTACCCAGGACACCAGCCTCCAGGACGGCTGCCTCATCTACAACGTCAAG ATCAGAGGGGTGAACTTCACATCCAACGGCCCTGTGATGCAGAAGAAAACACTCGGCTGG GAGGCCTTCACCGAGACGCTGTACCCCGCTGACGGCGGCCTGGAAGGCAGAAACGACAT GGCCCTGAAGCTCGTGGGCGGGAGCCATCTGATCGCAAACGCCAAGACCACATATAGATC CAAGAAACCCGCTAAGAACCTCAAGATGCCTGGCGTCTACTATGTGGACTACAGACTGGAA AGAATCAAGGAGGCCAACAACGAGACCTACGTCGAGCAGCACGAGGTGGCAGTGGCCAG ATACTGCGACCTCCCTAGCAAACTGGGGCACAAGCTTAATTGA. MCs were cultured at 60% confluence and were subjected to lentiviral transduction using a multiplicity of infection at 1.0 in OptiMEM containing 8 μg/mL protamine sulfate linker (Cat#P3369, Sigma-Aldrich, St. Louis, MO). Twenty-four hours later, lentiviral media was replaced with DMEM containing 10% FBS for an additional 48 hours and then exposed to Puromycin for selection of MCs with stable expression. Selected confluent cells were subsequently sub-cultured for downstream experiments.

Immunofluorescence. Cells were fixed with 4% paraformaldehyde (PFA) for 20 min. After permeabilization with 0.1% Triton X-100 (Cat#BP151-100, Fisher Scientific, Waltham, MA) in PBS for 15 minutes, cells were incubated with primary antibodies (alpha-smooth muscle actin, Cell Signaling Technology, Danvers, MA; Cat#14968) followed by rhodamine-labeled secondary antibodies (Jackson ImmunoResearch, West Grove, PA) for 1 h. Nuclei were counterstained

with DAPI and mounted using ProLong™ Diamond Antifade Mountant (ThermoFisher Scientific; Cat#P36961). Cells were visualized by a Nikon i90 fluorescence microscope and images captured with NIS-Elements v2.0 (Nikon, Melville, NY).

3D-collagen gel contractility assay. As previously described (2), collagen gels were prepared by mixing one part of 3 mg/mL neutralized rat tail collagen type I (Gibco Life Technologies, Carlsbad, CA; Cat# A10483-01) with two parts of cell suspension in serum-free media. Cell suspensions at a density of 0.2 million cells per mL were seeded onto 24-well cell culture plates and the gels were allowed to polymerize at 37°C for 1h, before adding 1 ml of media. Polymerized gels were incubated overnight, and then the edges were gently detached from the walls of the well using a sterile spatula. After treatments, the gels were photographed and the images were measured for gel area using the NIH ImageJ 1.54g software.

BrdU Incorporation. As previously described (3), MCs were plated at a density of 10,000 cells in 96-well cell culture plates, and BrdU labeling protocol was followed as per manufacturer's instructions (Cell Biolabs, Inc., San Diego, CA; Cat#CBA-251). Briefly, cells were quiesced overnight and then labeled with BrdU for 6h at 37ºC. Cells were rinsed with PBS and then fixed with fixative/denaturing solution for 0.5h at 37ºC. Cells were rinsed with PBS and subjected to protein block for 1h at room temperature, followed by incubation with anti-BrdU antibody and HRP-conjugated secondary antibody for 1h each. Cells were rinsed with PBS and then incubated with the substrate solution in the dark for 15 min followed by addition of the "stop solution". Absorbance was read on an ELISA plate reader at 450nm wavelength.

Wound-healing 'scratch' assay. As previously described (4), MCs were seeded in 24-well plates and cultured to 90% confluence. The cells were quiesced overnight and then wounded by scratching with a pipette tip. Cells were observed for 72h, after which cells were PFA-fixed, and nuclei were counterstained with DAPI and mounted using ProLong™ Diamond Antifade Mountant (ThermoFisher Scientific; Cat#P36961). Images of wound closure was captured at 40x using NIS-Elements v2.0 (Nikon, Melville, NY), and the blue fluorescence intensity of the

DAPI-stained infiltrating cells within a fixed area of the wound was measured using the NIH ImageJ 1.54g software.

Supplemental Figures

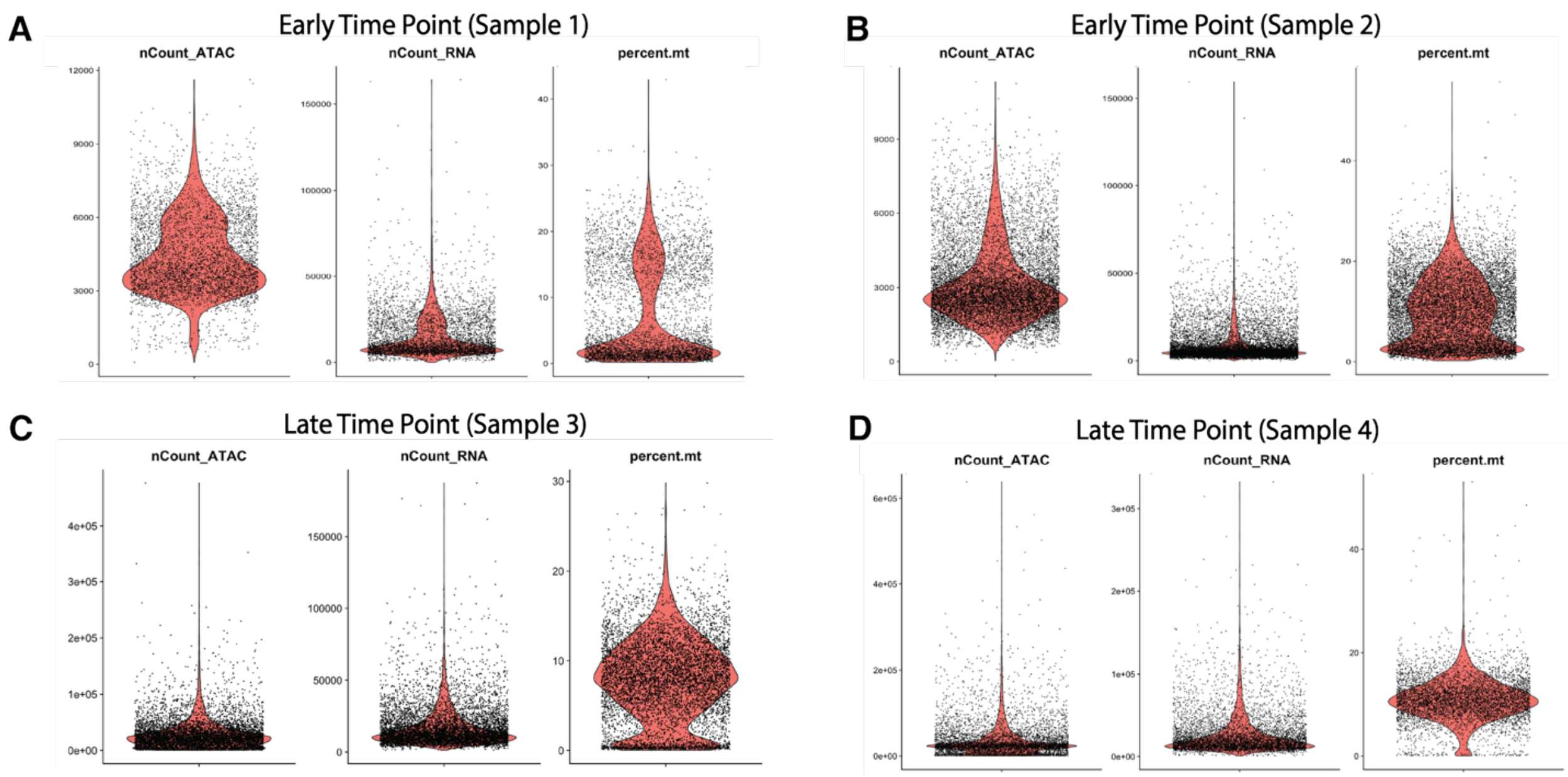

Supplemental Figure 1. Single-cell multiome quality control metrics.

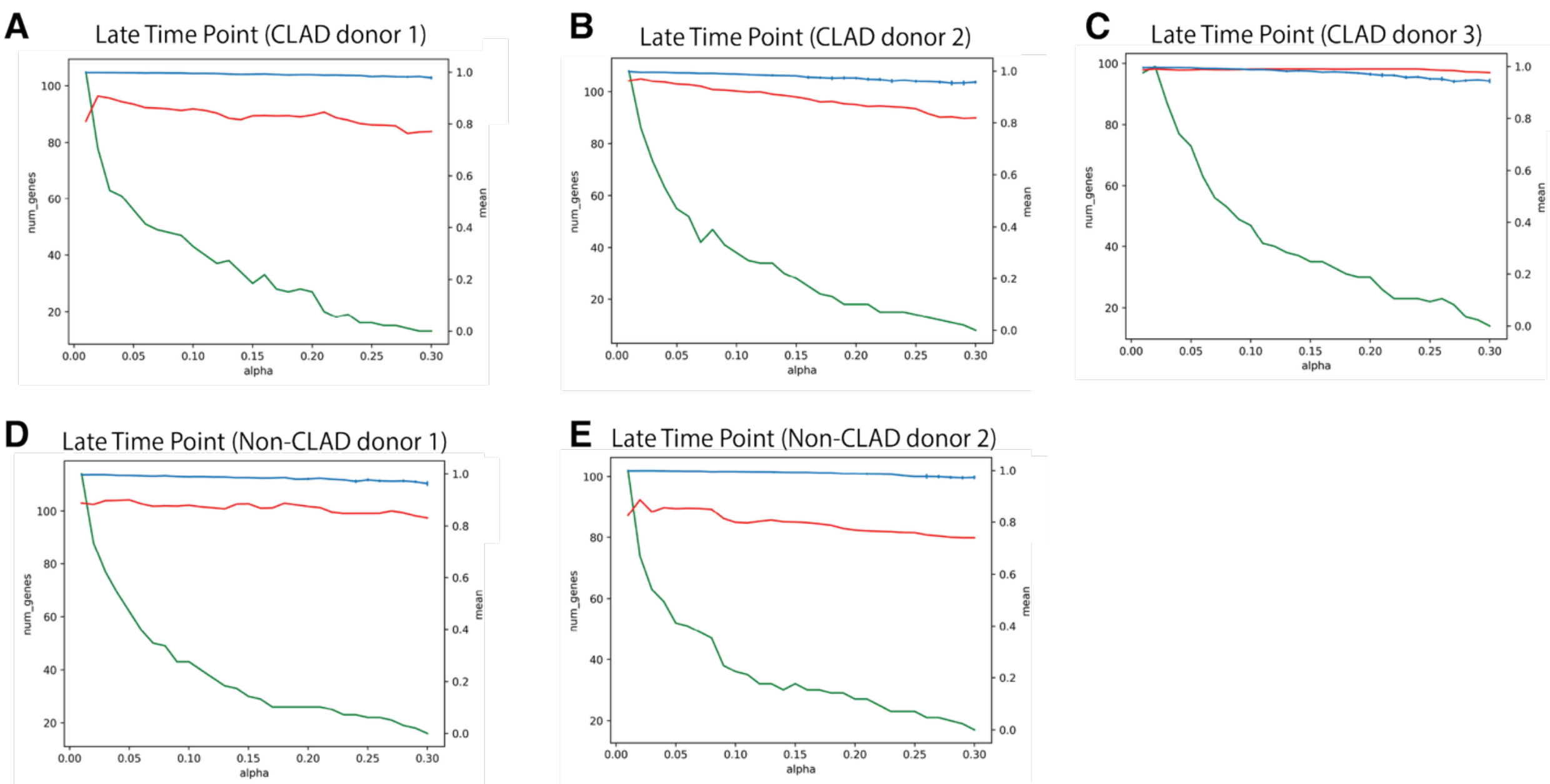

Supplemental Figure 2. Performance of logistic regression models on cells from held-out donors. The blue line represents the average accuracy of the logistic regression classifiers, calculated using 5-fold cross-validation, with error bars indicating the variance. The green line shows the number of genes, determined by counting the non-zero coefficients from the logistic regression classifiers trained on the test sets. The red line represents the accuracy of the logistic regression classifiers when predicting cells from the held-out donor.

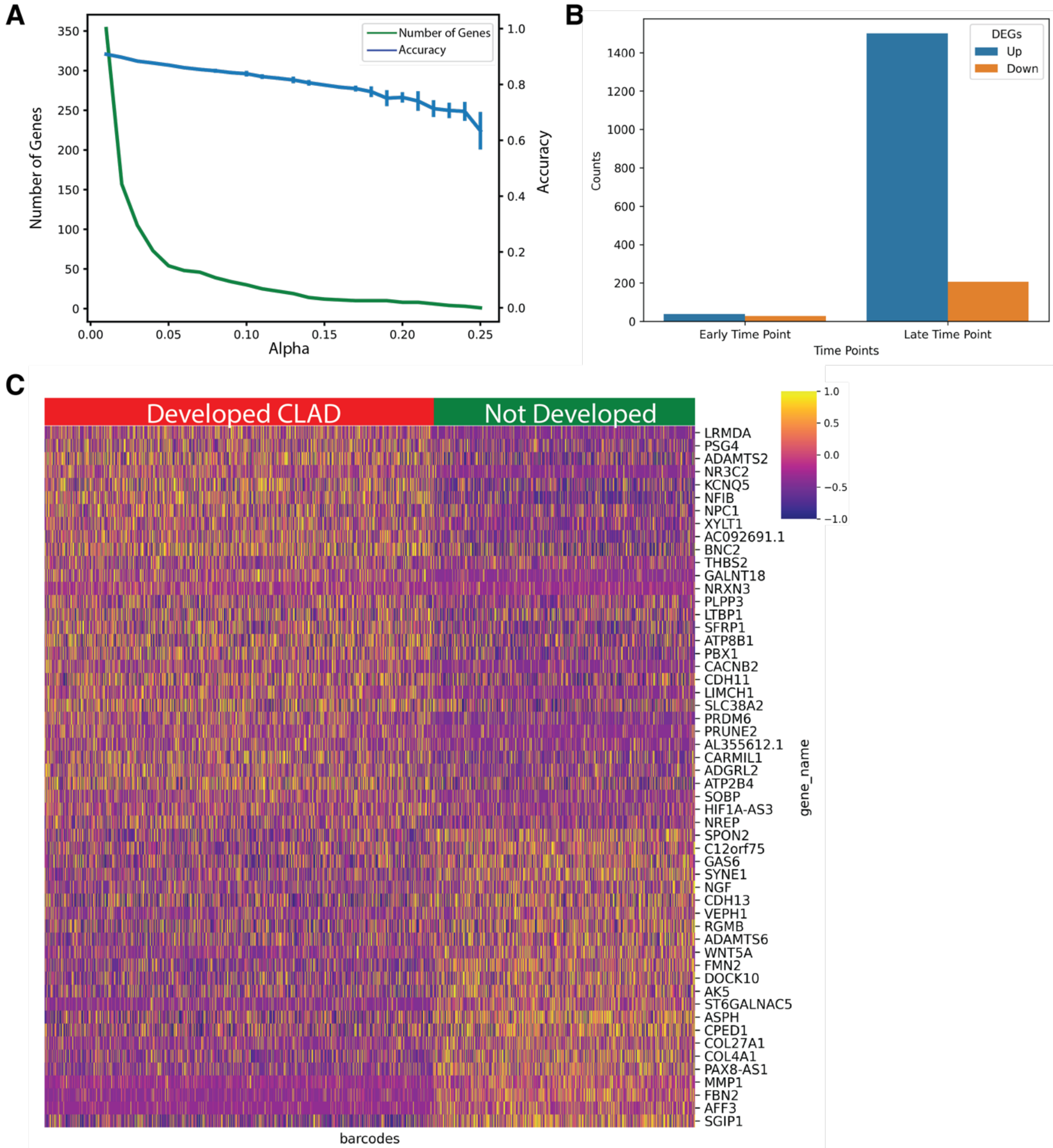

Supplemental Figure 3. Classifying early time point MCs as developed CLAD vs. not developed CLAD from single-cell gene expression. (A) The blue lines indicate average accuracy score of the logistic regression classifiers from 5-fold cross-validation. Error bars indicate the variance in accuracy across folds. The green lines indicate the number of genes with non-zero coefficients from the logistic regression classifiers trained on the test sets. **(B)** The number of differentially expressed genes in early-time-point and late-time-point MCs. Blue bars indicate the upregulated. **(C)** Heatmaps depicting the expression levels of gene signatures in the early time-point MCs constructed from the logistic regression classifiers with α of 0.05. Each column represents a cell, and each row represents one gene colored by gene expression level.

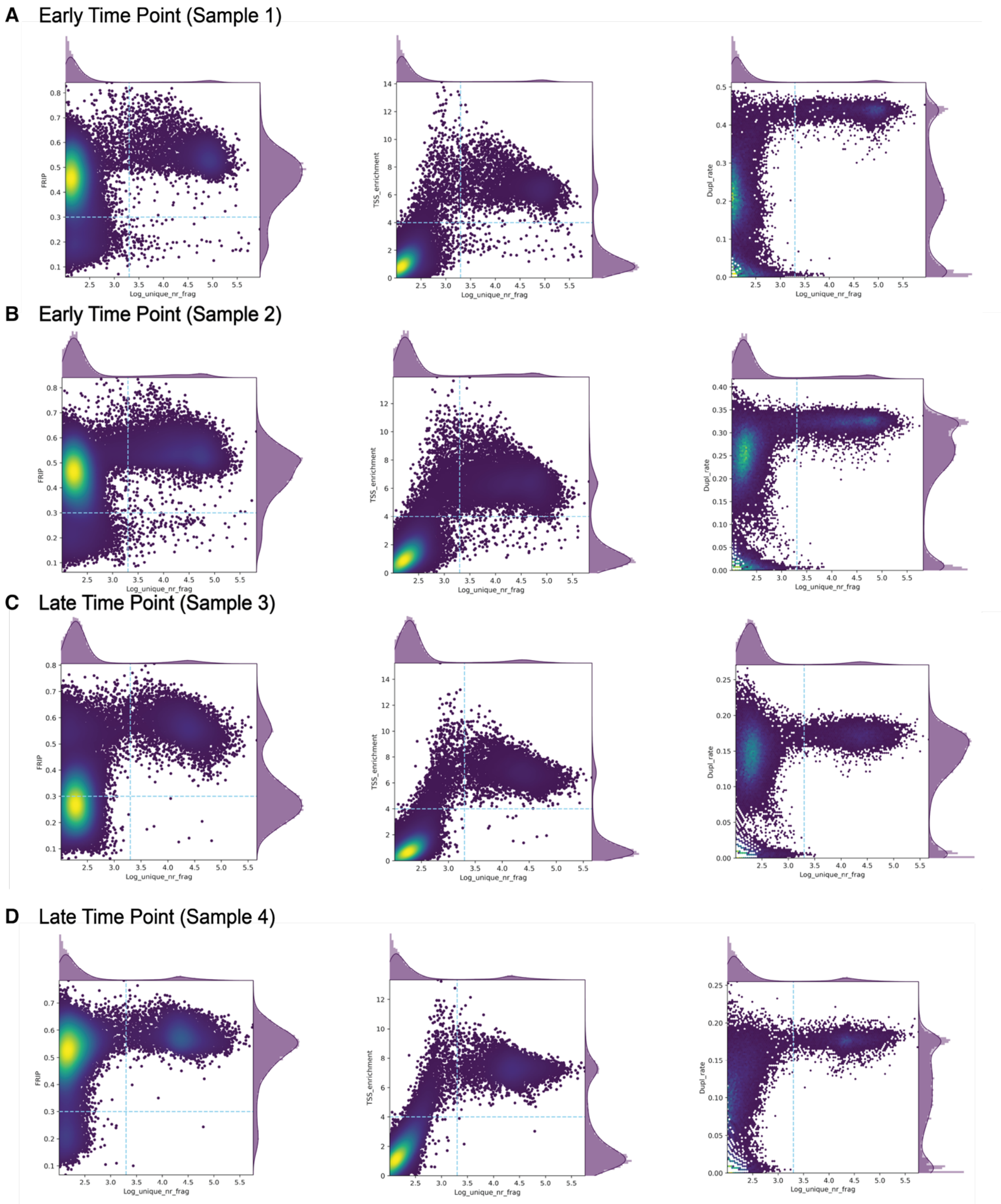

Supplemental Figure 4. Barcode-level scATAC-seq quality statistics. The x-axis is the log number of unique fragments. The y-axis in the first plot of each sample is Fraction of Reads In Peaks (FRIP). The y-axis in the second plot of each sample is Transcription Start Site (TSS) enrichment score. The y-axis in the third plot of each sample is duplication rate.

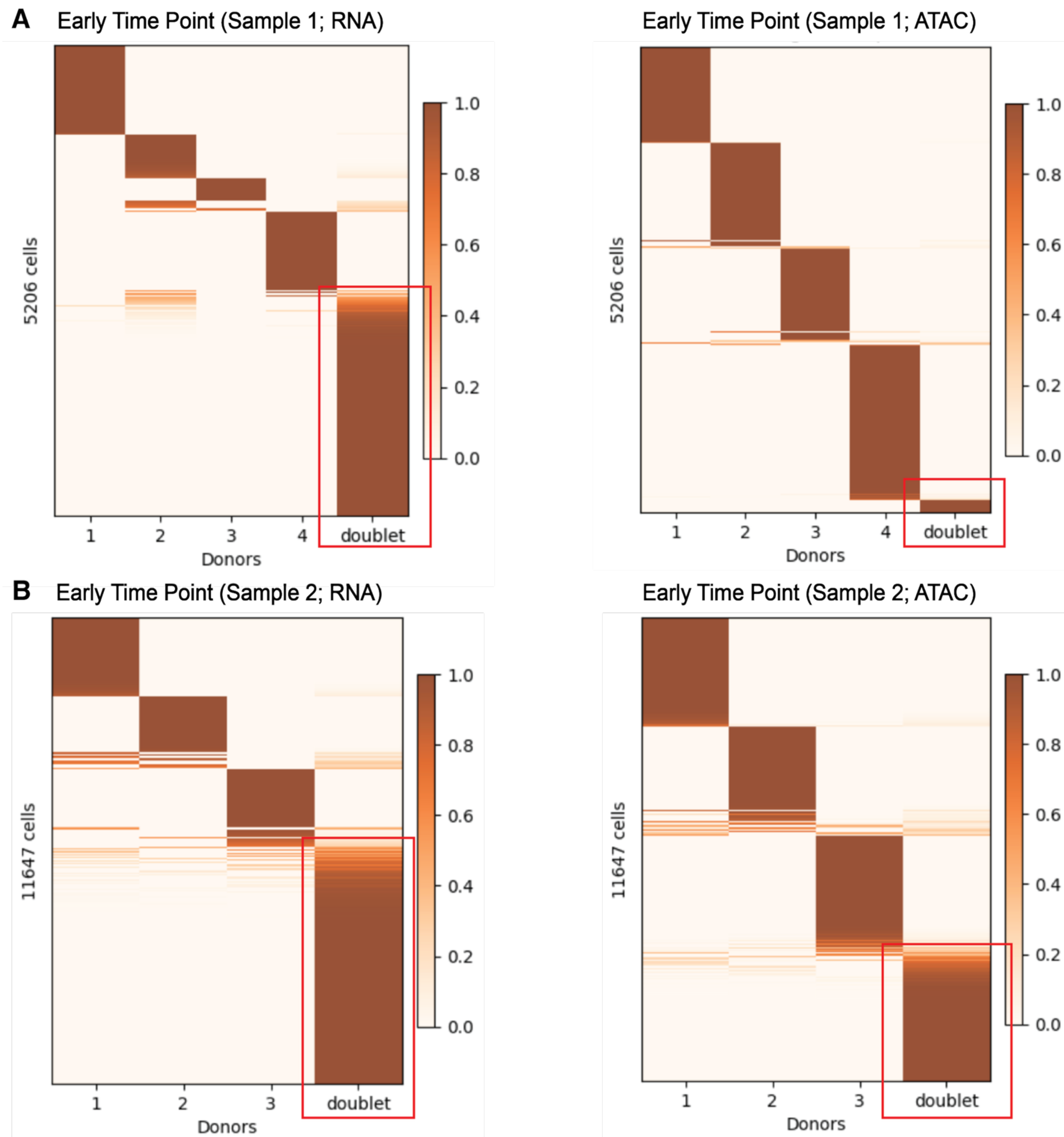

Supplemental Figure 5. Cell assignment from vireo for the early time point MCs, developed CLAD (A) and not developed CLAD (B). The left plot of each is from the RNA modality and the right plot is from the ATAC modality. The x-axis is the donor id and each line in the y-axis is a cell colored by the probability of assignment to the donor ID. Cells predicted to be doublets by either RNA or ATAC genotype are indicated with a red box.

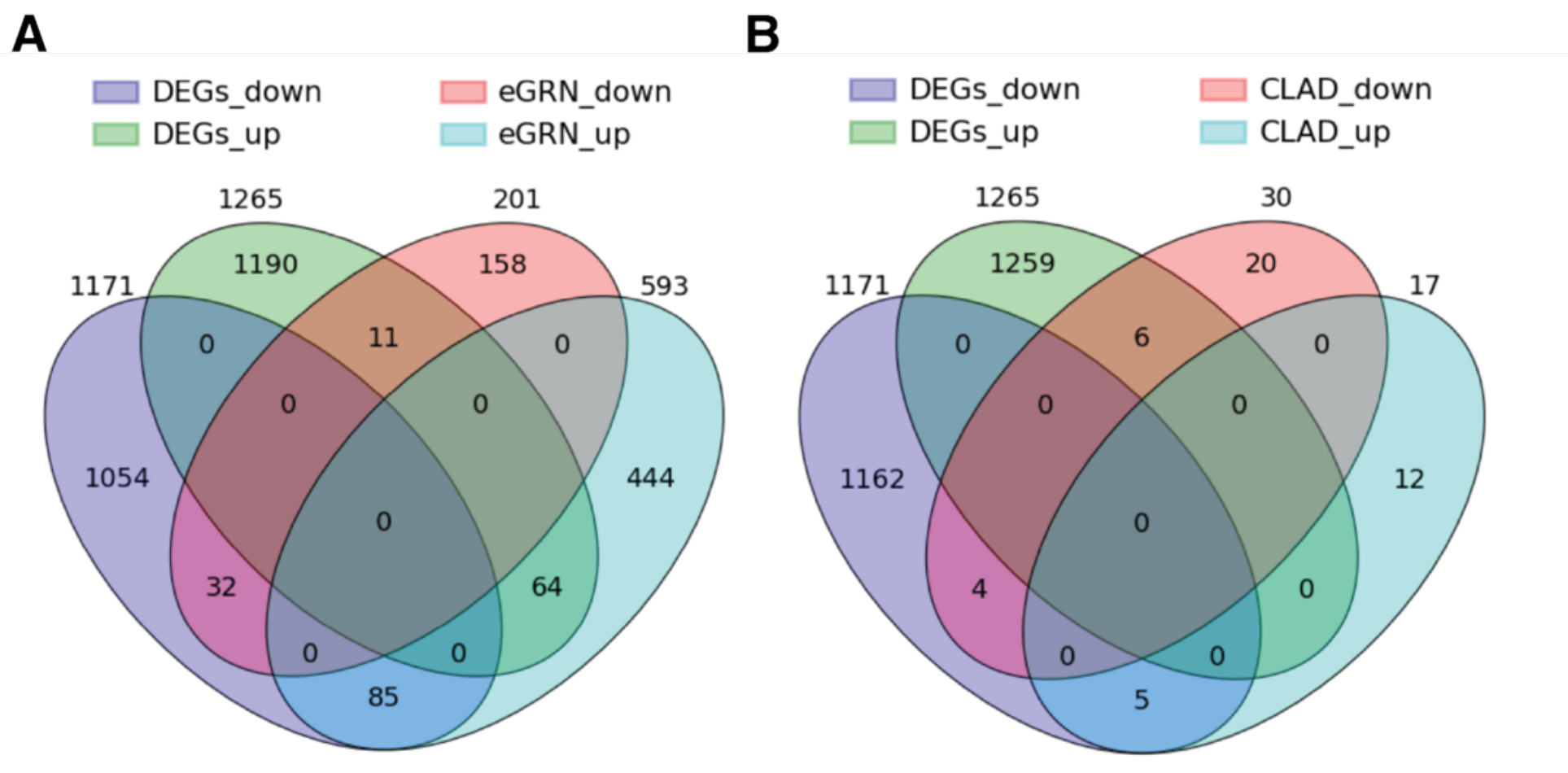

Supplemental Figure 6. Differentially expressed genes in CEBPD knockdown experinmet compared to eGRN predicted targets and CLAD gene signatures. (A) Venn diagram showing the overlap between differentially expressed genes following *CEBPD* silencing and predicted CEBPD targets from the eGRN. **(B)** Venn diagram showing the overlap between differentially expressed genes following *CEBPD* silencing and CLAD gene signatures from **Figure 2E**.

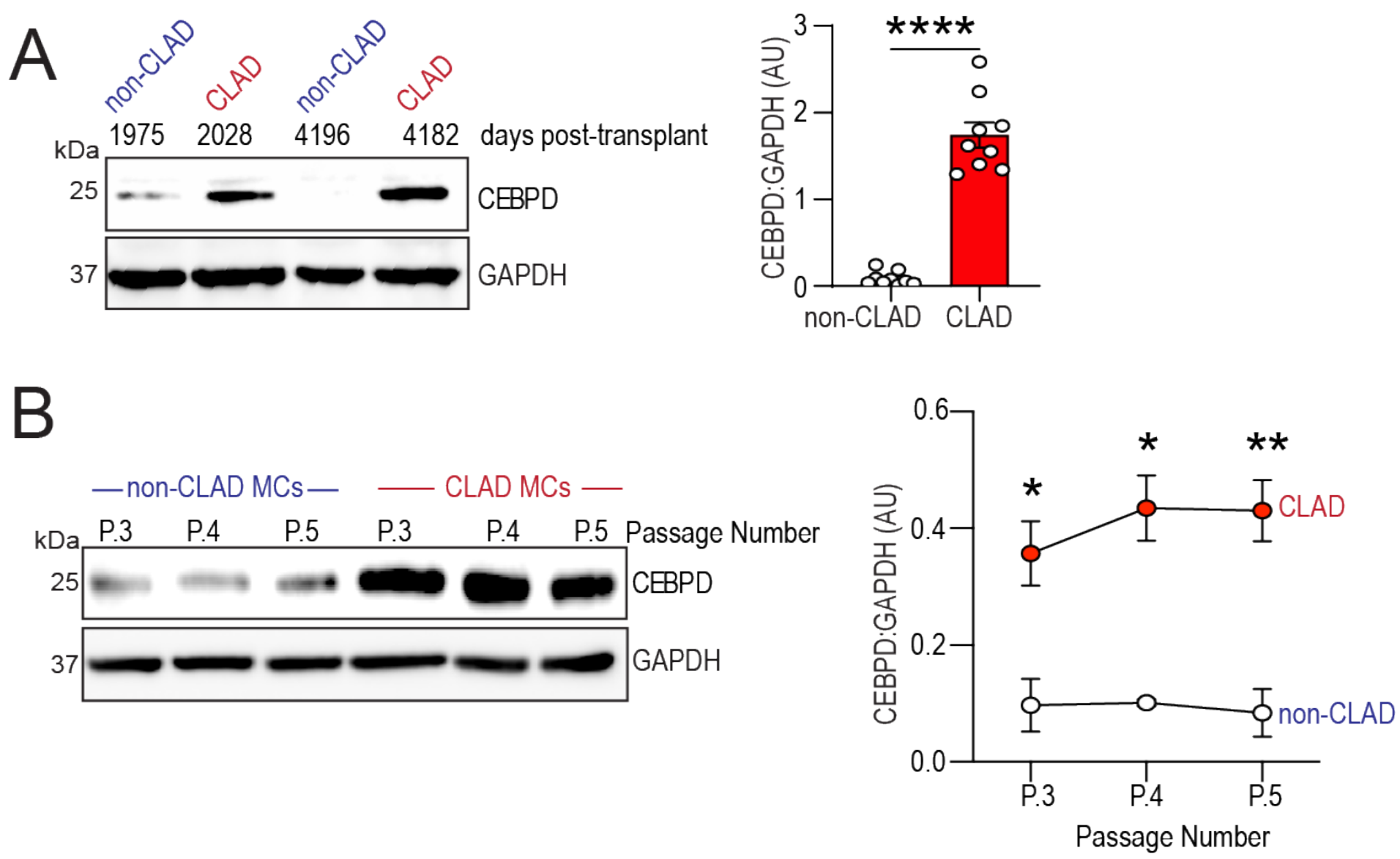

Supplemental Figure 7. CLAD MCs display stable upregulation of CEBPD across time post-transplant and passages in vitro. (A) CLAD MCs were matched to non-CLAD MCs by time post-transplantation (Supplemental Table 2). Protein samples from whole cell lysates were evaluated for CEBPD by Western Blot. Representative immunoblotting images and densitometry analysis are shown. (B) Non-CLAD and CLAD MCs were passaged over time and protein expression of CEBPD from whole cell lysates was evaluated by Western Blot. Densitometry of CEBPD expression normalized to GAPDH. Data are represented as means ± SEM. For each group, (A) n=9 (B) n=3. *, $P < 0.05$; **, $P < 0.01$; ****, $P < 0.0001$. Statistics: (A) paired t-test, (B) 2-way ANOVA; *post hoc* test: Bonferroni's test.

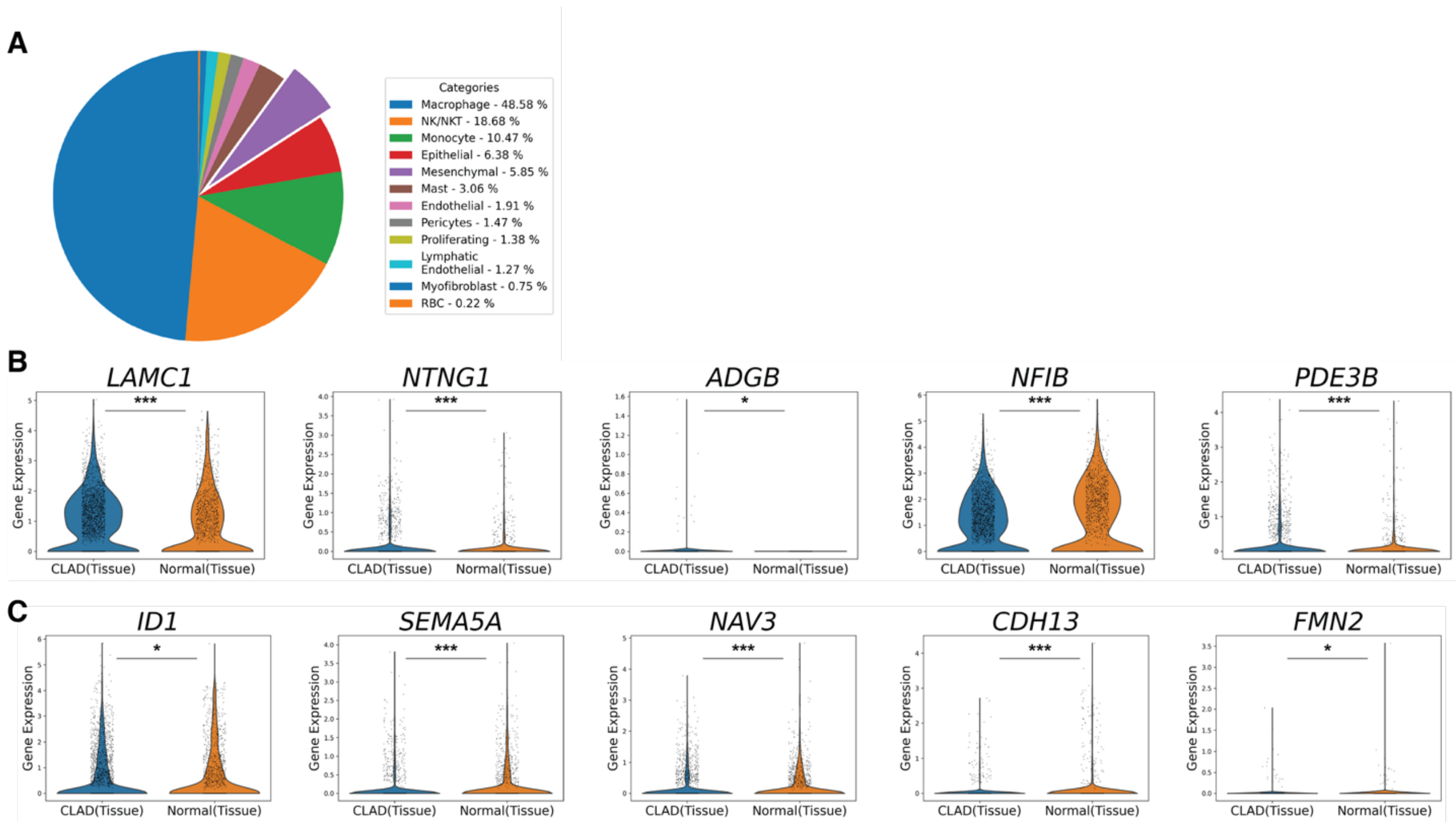

Supplemental Figure 8. scRNA-seq analysis between CLAD lung autopsy samples and healthy controls. (A) Pie chart showing the proportional distribution of each cell-type within non-depleted human lung autopsy samples. **(B)** Violin plots depicting the expression levels of *LAMC1*, *NTNG1*, *ADGB*, *NFIB*, *and PDE3B* across conditions. **(C)** Violin plots depicting the expression levels of *ID1*, *SEMA5A*, *NAV3*, *CDH13*, *and FMN2* across conditions. (*p<0.05; ***p ≤ 0.001)

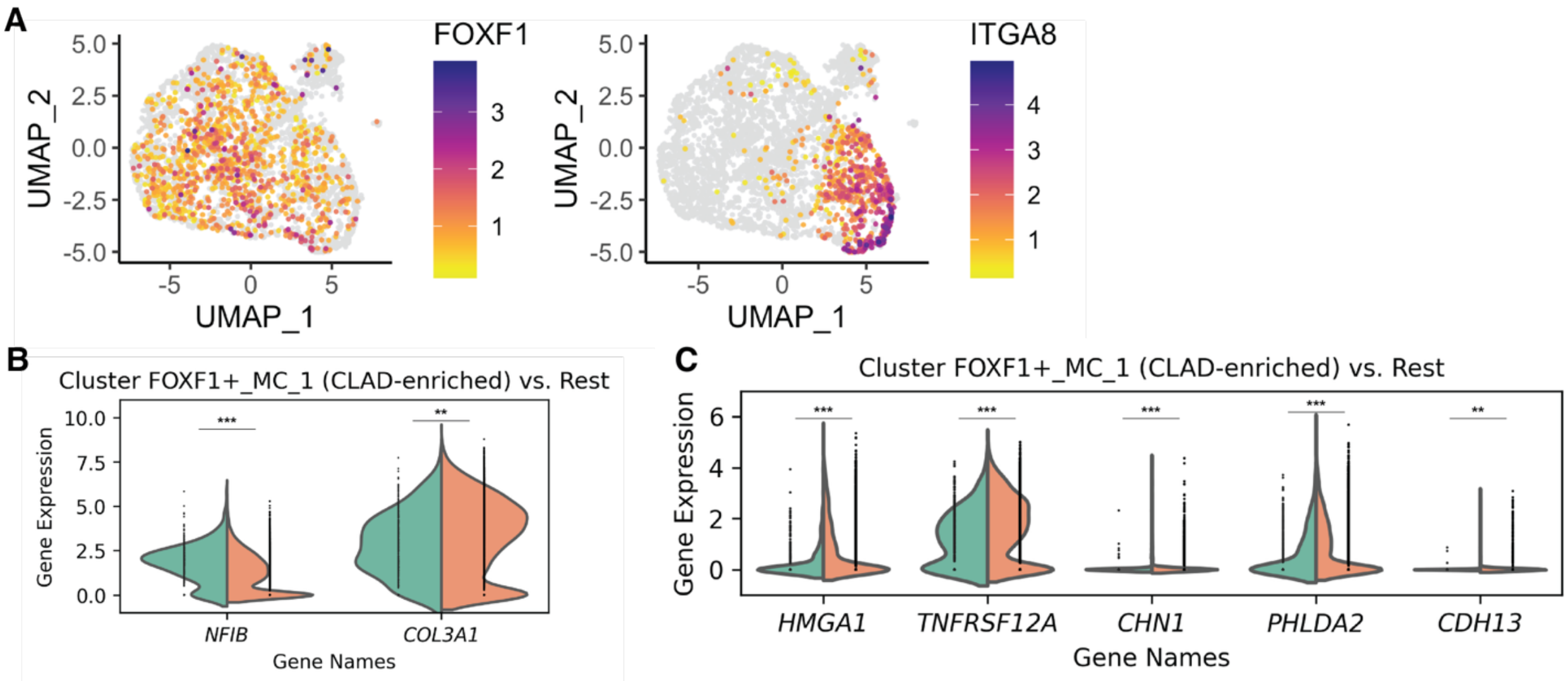

Supplemental Figure 9. scRNA-seq analysis of tissue MCs. (A,B) Split violin plots showing the expression levels of *NFIB* and *COL3A1*. Each violin is split to compare cluster FOXF1+_MC_1 (CLAD-enriched) with the rest of the MCs, with the width representing the distribution of expression values. **(C)** Split violin plots showing the expression levels of *HMGA1*, *TNFRSF12A*, *CHN1*, *PHLDA2*, and *CDH13*. (**p ≤ 0.01; ***p ≤ 0.001)

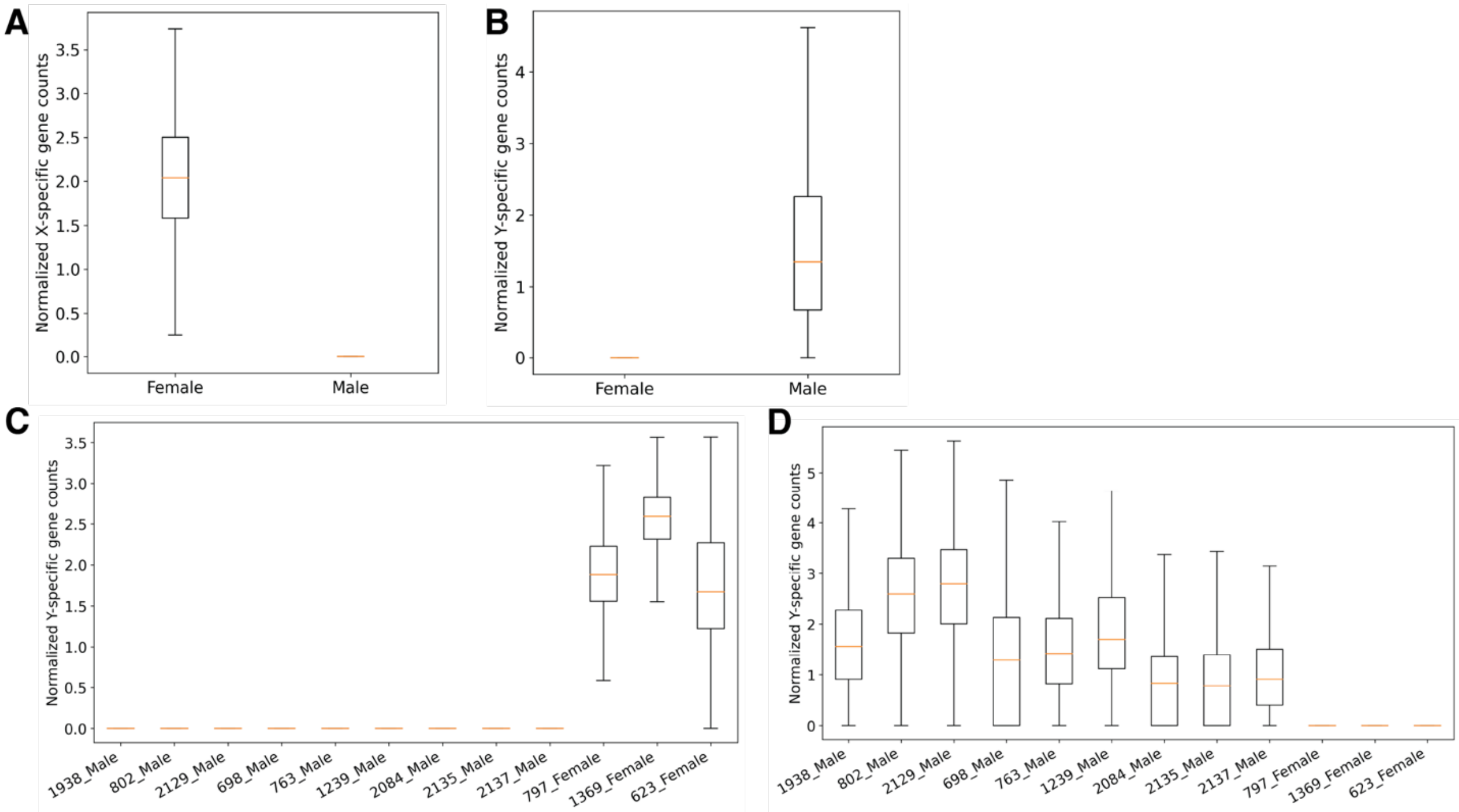

Supplemental Figure 10. Sex-chromosome gene expression in cultured MCs. (A,B) Boxplots showing normalized expression of X-linked **(A)** and Y-linked **(B)** genes stratified by donor sex. The x-axis is the normalized expression of sex-linked genes. The y-axis is the donor gender. (**C,D**) Same analysis is shown at the individual donor level.

Supplemental Tables

Characteristic	CLAD (n=20)	Control (n=24)	Total (n=44)	P-Value
Age in years at Transplant, mean (sd)	45.6 (16.2)	54.1 (9.6)	50.2 (13.5)	0.04
Female, n(%)	9 (45)	6 (25)	15 (35)	0.16
Pre-Transplant Diagnosis, n(%)				
COPD	5 (25.0)	9 (37.5)	14 (31.8)	0.10
Cystic Fibrosis	4 (20.0)	2 (8.3)	6 (13.6)	
ILD	6 (30.0)	12 (50.0)	18 (40.9)	
Other	5 (25.0)	1 (4.2)	6 (13.6)	
BAL Characteristics, mean (sd)				
Volume (mL)	19.9 (6.4)	18.9 (7.5)	19.3 (6.9)	0.64
Cell Count (x10^6)	3.2 (1.4)	3.2 (3.8)	3.2 (2.9)	1.00
Red Blood Cells, cells/µL	3318 (3669)	1955 (1952)	2589 (2925)	0.12
White Blood Cells, cells/µL	1143 (891)	570 (481)	836 (751)	0.01
Lymphocytes, cells/µL	8.2 (6.9)	5.7 (8.0)	6.8 (7.5)	0.28
Neutrophils, cells/µL	33.5 (23.6)	19.9 (24.2)	26.2 (24.6)	0.07
Histiocytes, cells/µL	57.7 (21.5)	73.7 (26.3)	66.3 (25.2)	0.03
Eosinophils, cells/µL	0.7 (1.3)	0.5 (1.0)	0.6 (1.1)	0.57
Time from Transplant to CLAD onset in days, mean (sd)	1171 (613)	-	-	-
Time from CLAD diagnosis to BAL, mean(sd)	388 (671)	-	-	-
CLAD Phenotype, n(%)				
BOS	8 (40)	-	-	-
RAS	6 (30)	-	-	-
Mixed	2 (10)	-	-	-
Undefined	4 (20)	-	-	-

Supplemental Table 1. Patient demographics and transplant statistics

Time-matched samples	Time Post-Txp	Difference in Days between Pairs	CLAD Phenotype
Time-matched CLAD 1	263	89	RAS
Time-matched Non-CLAD 1	352		-
Time-matched CLAD 2	305	55	BOS
Time-matched Non-CLAD 2	360		-
Time-matched CLAD 3	309	121	BOS
Time-matched Non-CLAD 3	430		-
Time-matched CLAD 4	815	115	BOS
Time-matched Non-CLAD 4	930		-
Time-matched CLAD 5	935	65	RAS
Time-matched Non-CLAD 5	1000		-
Time-matched CLAD 6	1047	36	Undefined/Mixed
Time-matched Non-CLAD 6	1011		-
Time-matched CLAD 7	1609	34	RAS
Time-matched Non-CLAD 7	1575		-
Time-matched CLAD 8	2028	53	Undefined/Mixed
Time-matched Non-CLAD 8	1975		-
Time-matched CLAD 9	4182	14	BOS
Time-matched Non-CLAD 9	4196		-
All BALs in the matched pairs dataset were isolated from patients within 4 months (+/- 1 day) of each other. Difference between matched pairs and their corresponding days-post transplant are listed in column D.			

Supplemental Table 2. Time-matched CLAD and non-CLAD samples with post-transplant timing and CLAD phenotypes

Reference

1. Lu L, Welch JD. PyLiger: scalable single-cell multi-omic data integration in Python. *Bioinformatics*. 2022;38(10):2946–2948.

2. Hecker L, et al. NADPH oxidase-4 mediates myofibroblast activation and fibrogenic responses to lung injury. *Nat Med*. 2009;15(9):1077–1081.

3. Vittal R, et al. Effects of the protein kinase inhibitor, imatinib mesylate, on epithelial/mesenchymal phenotypes: implications for treatment of fibrotic diseases. *J Pharmacol Exp Ther*. 2007;321(1):35–44.

4. Vittal R, et al. IL-17 induces type V collagen overexpression and EMT via TGF-β-dependent pathways in obliterative bronchiolitis. *Am J Physiol Lung Cell Mol Physiol*. 2013;304(6):L401-14.
