# Supplementary material for: Single-cell multiomic analysis of mesenchymal cells reveals molecular signatures and regulators of lung allograft fibrosis
Source: JCI Insight. 2026 Apr 23;11(13):e196651. doi: 10.1172/jci.insight.196651 (PMC13460852; doi:10.1172/jci.insight.196651)

Figure 6B.

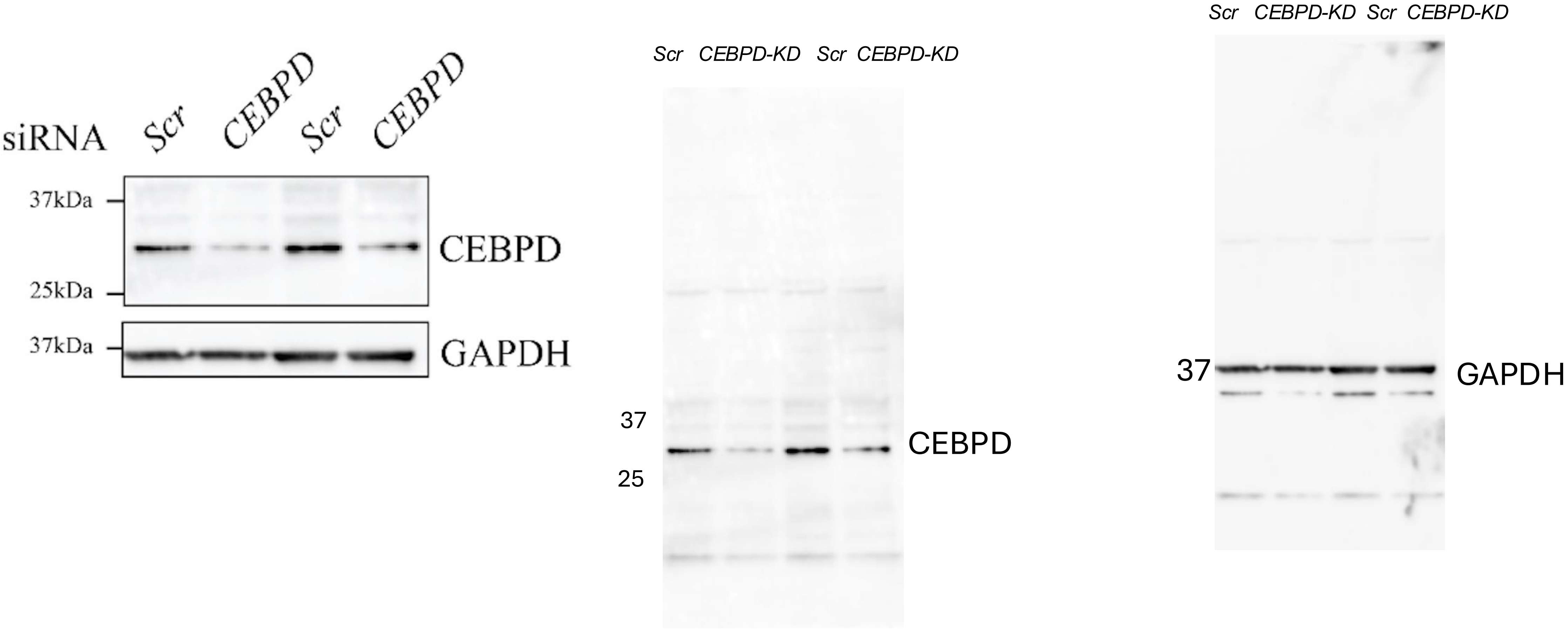
siRNA
Scr
CEBPD
Scr
CEBPD
37kDa
CEBPD
25kDa
37kDa
GAPDH
Scr CEBPD-KD Scr CEBPD-KD
37
CEBPD
25
Scr CEBPD-KD Scr CEBPD-KD
37
GAPDH

Figure 7A.

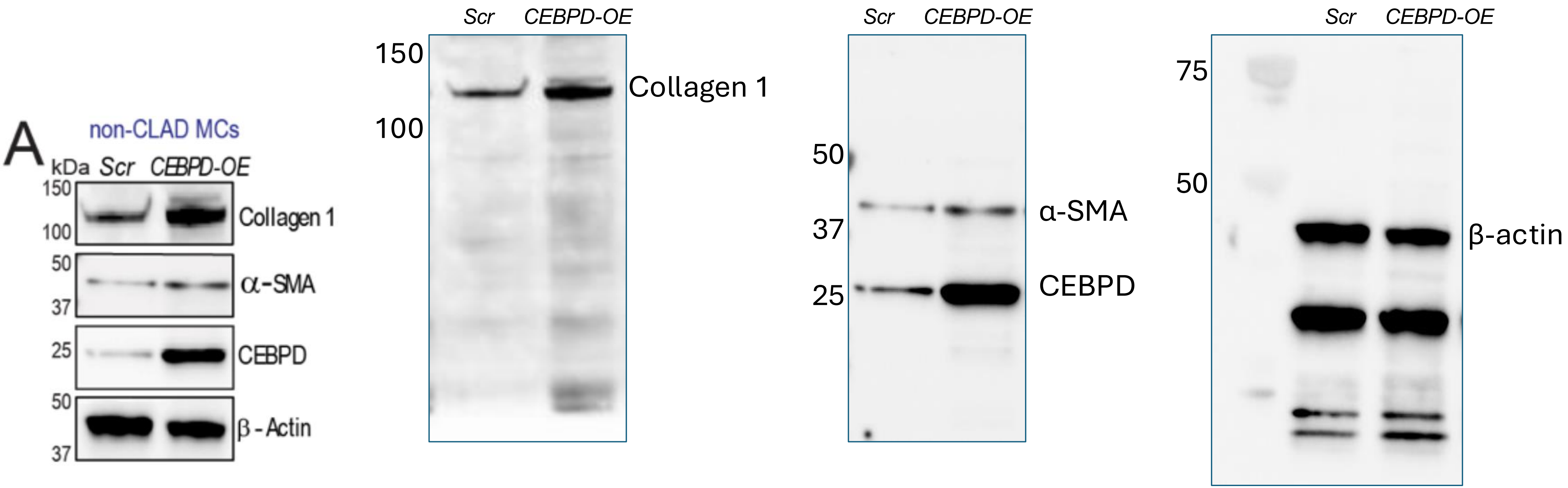

A
non-CLAD MCs
kDa
Scr
CEBPD-OE
150
100
Collagen 1
50
37
α-SMA
25
CEBPD
50
37
β-Actin
Scr
CEBPD-OE
150
100
Collagen 1
Scr
CEBPD-OE
50
37
25
α-SMA
CEBPD
Scr
CEBPD-OE
75
50
β-actin

Figure 7B.

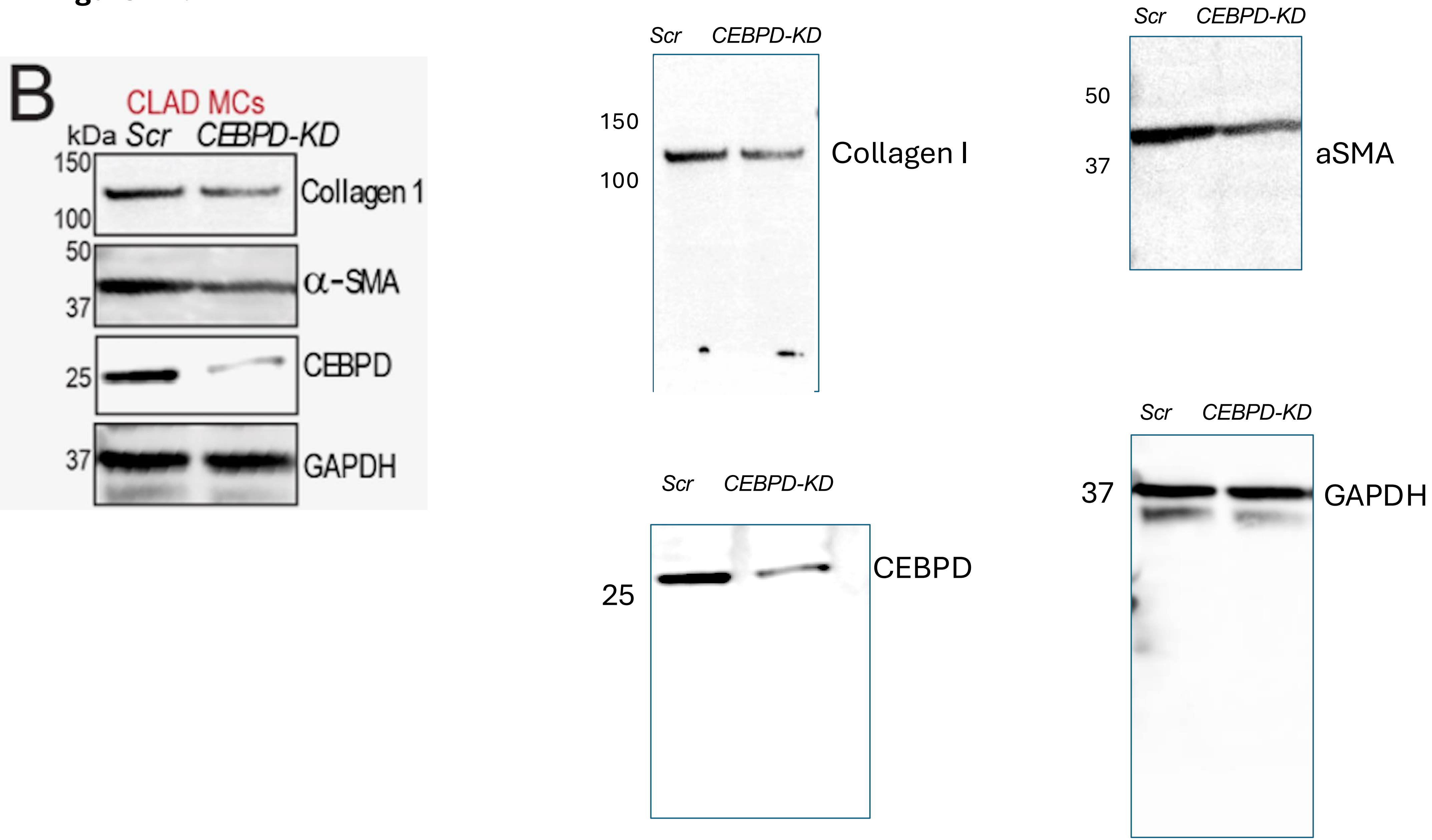
B
CLAD MCs
kDa
Scr
CEBPD-KD
150
100
Collagen 1
50
37
α-SMA
25
CEBPD
37
GAPDH
Scr
CEBPD-KD
150
100
Collagen I
Scr
CEBPD-KD
50
37
aSMA
Scr
CEBPD-KD
25
CEBPD
Scr
CEBPD-KD
37
GAPDH

Figure 7E.

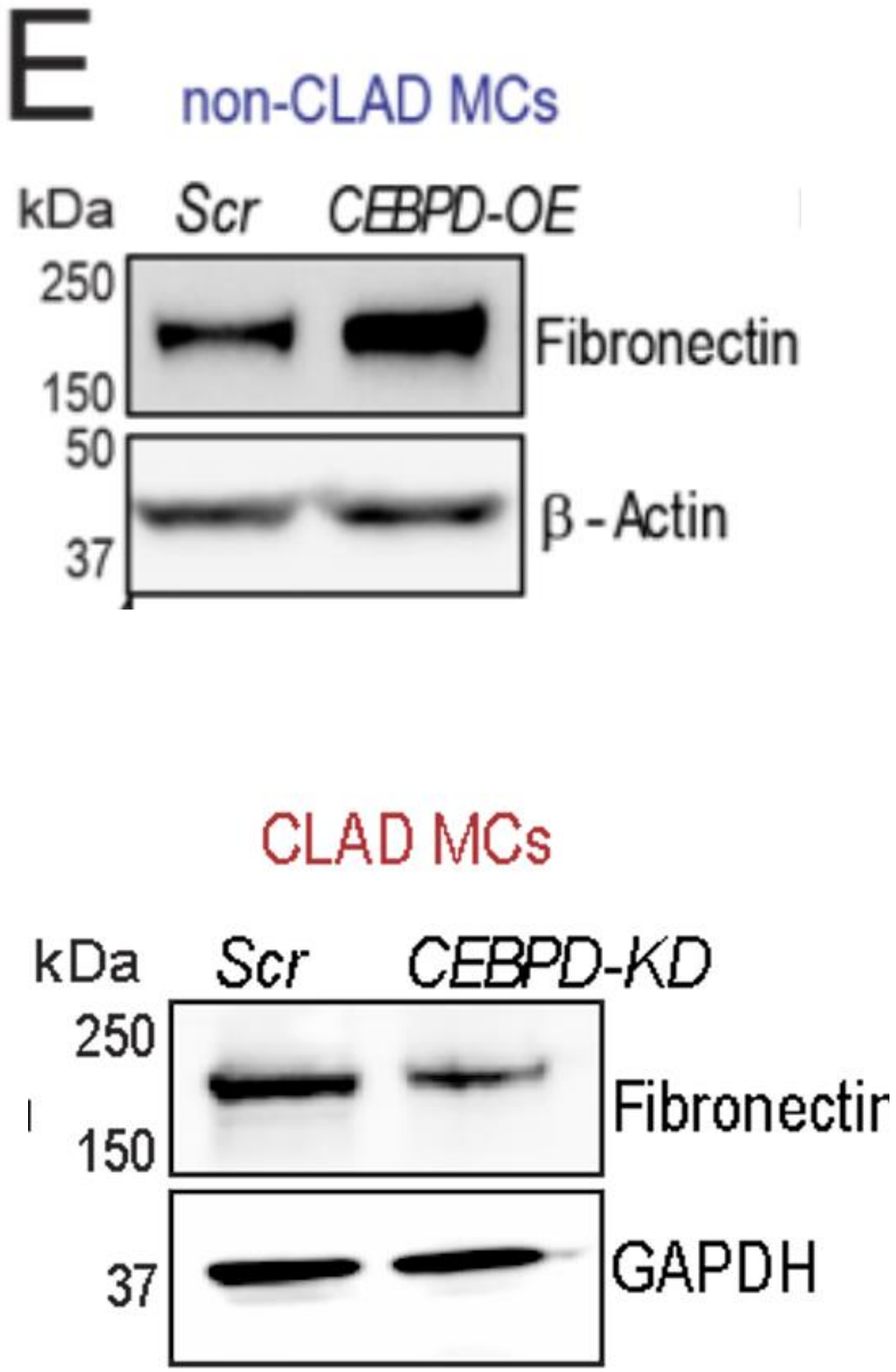
E
non-CLAD MCs
kDa
Scr
CEBPD-OE
250
150
Fibronectin
50
37
β-Actin
CLAD MCs
kDa
Scr
CEBPD-KD
250
150
Fibronectir
37
GAPDH

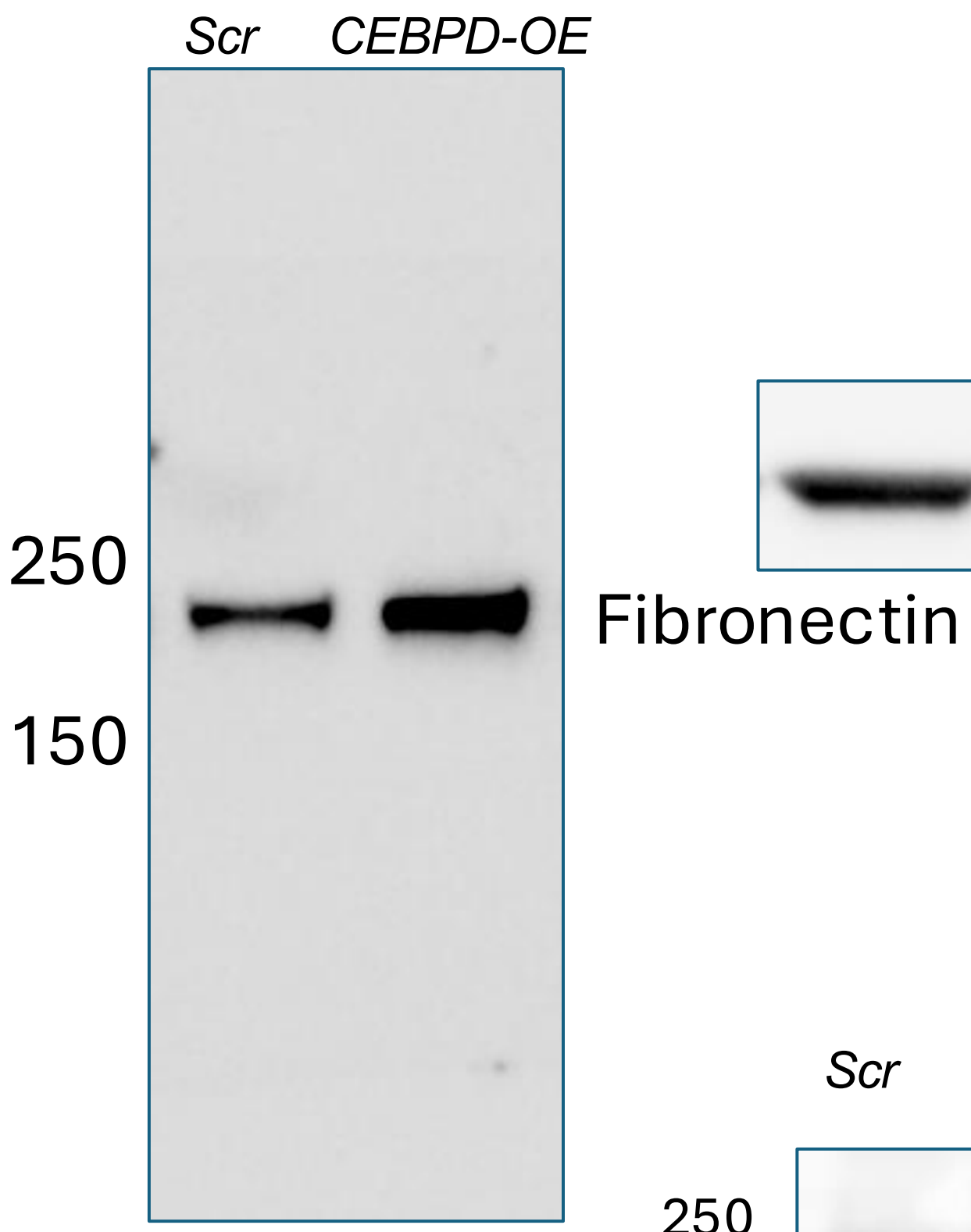
Scr
CEBPD-OE
250
150
Fibronectin

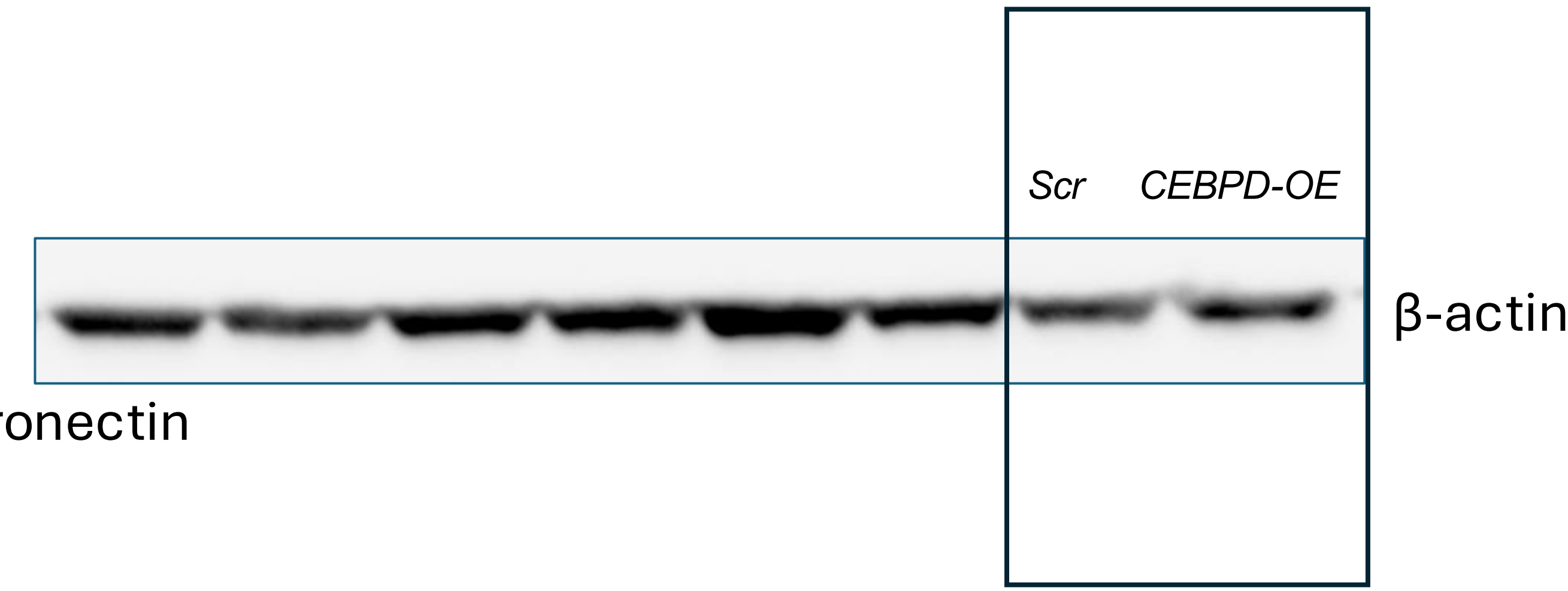
Scr
CEBPD-OE
β-actin

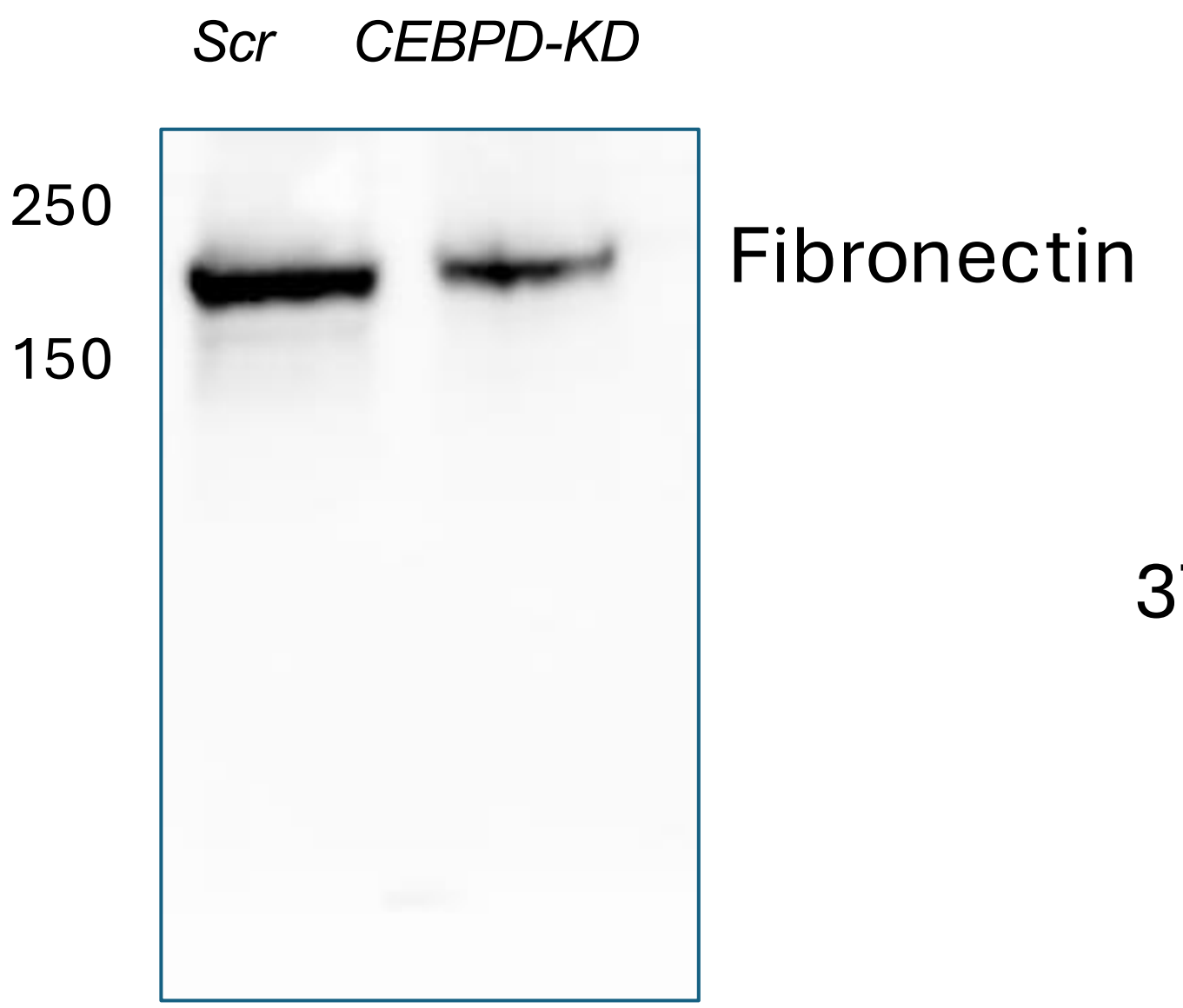
Scr
CEBPD-KD
250
150
Fibronectin

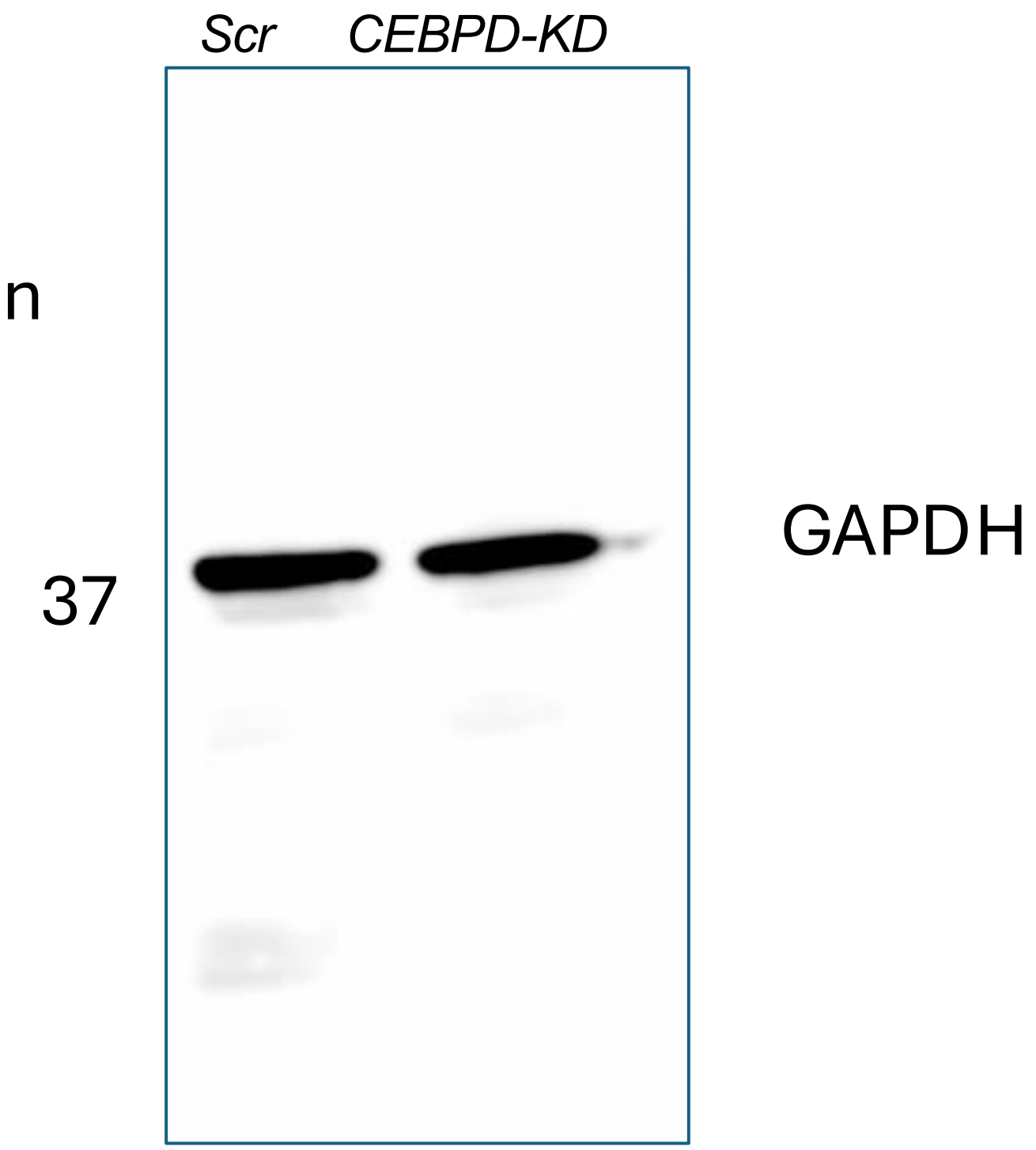
Scr
CEBPD-KD
37
GAPDH

Figure 7H.

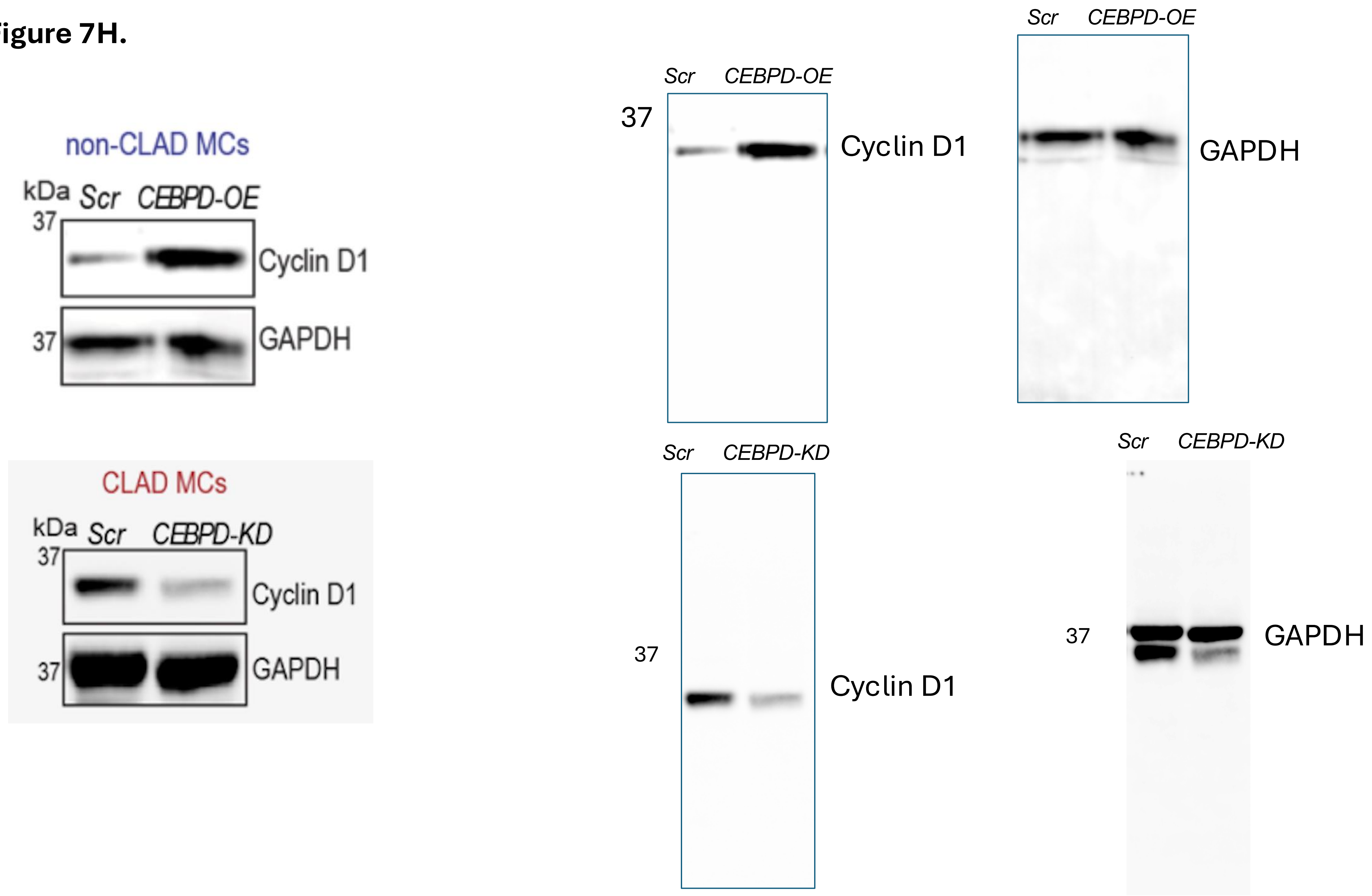

non-CLAD MCs
kDa
Scr
CEBPD-OE
37
Cyclin D1
37
GAPDH
CLAD MCs
kDa
Scr
CEBPD-KD
37
Cyclin D1
37
GAPDH
Scr
CEBPD-OE
37
Cyclin D1
Scr
CEBPD-OE
GAPDH
Scr
CEBPD-KD
37
Cyclin D1
Scr
CEBPD-KD
37
GAPDH

Supplemental Figure 7A

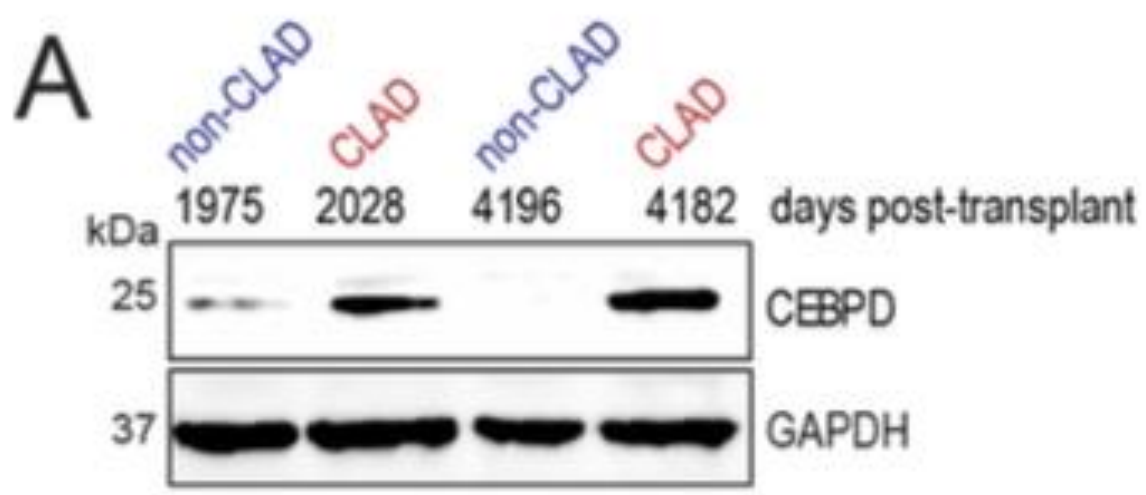

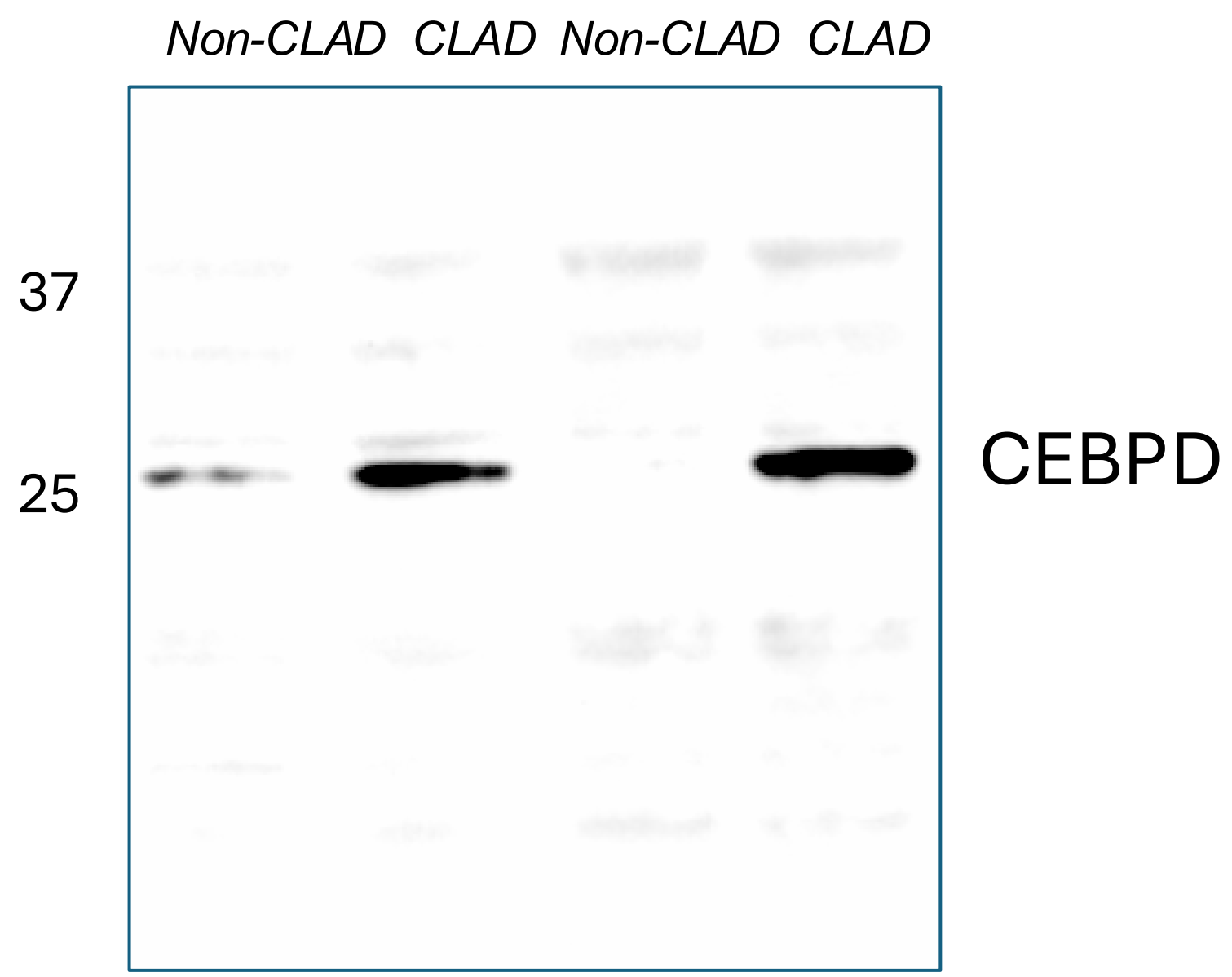

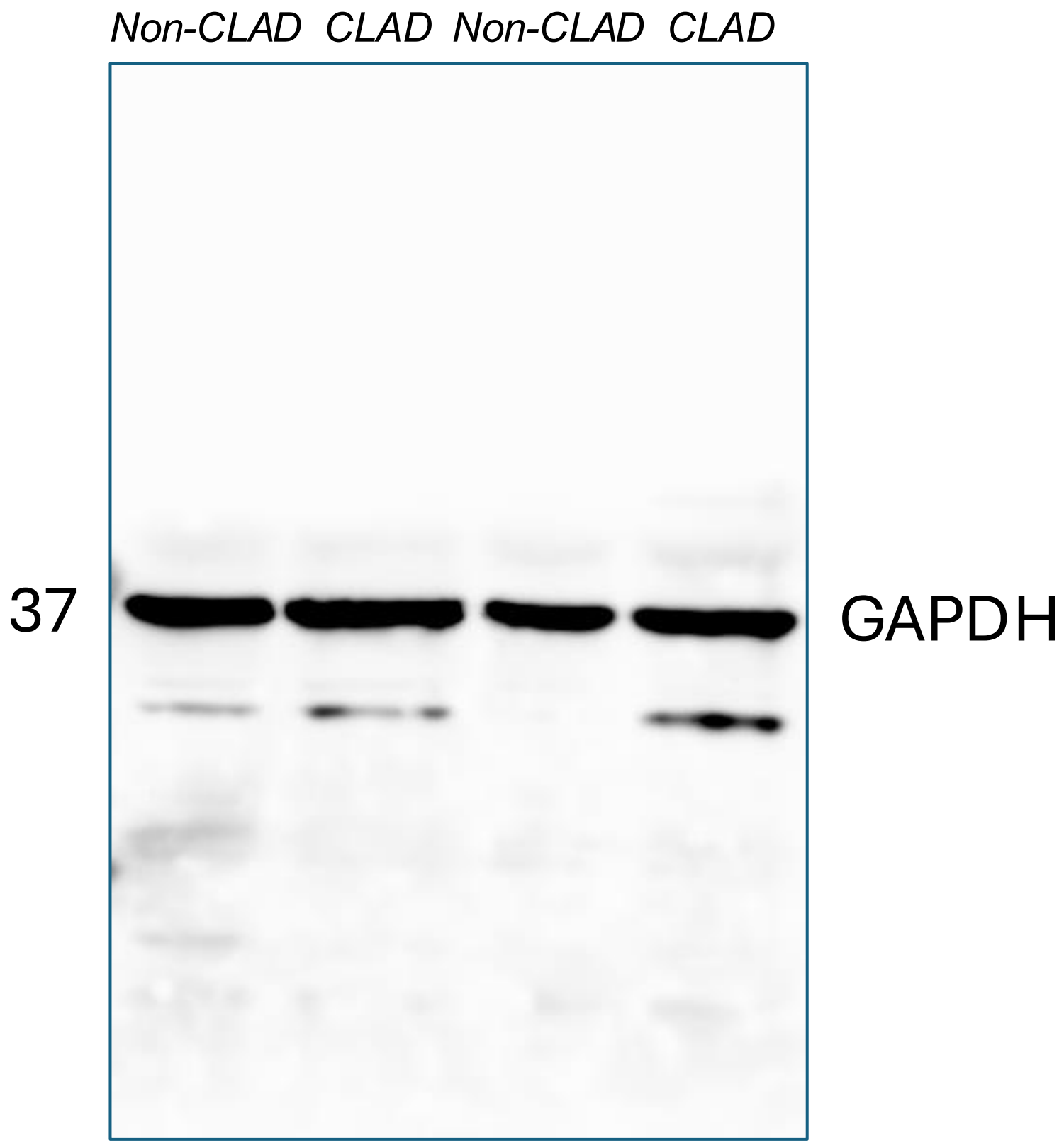

Supplemental Figure 7B

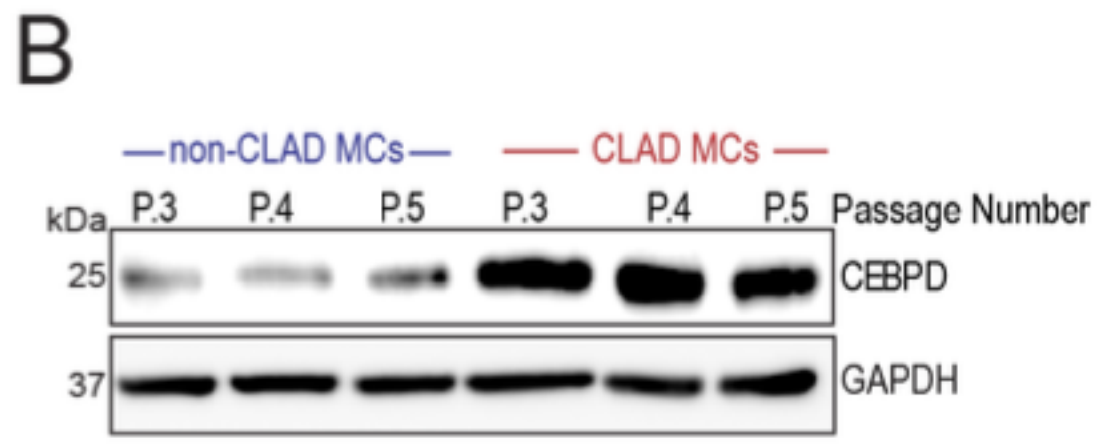

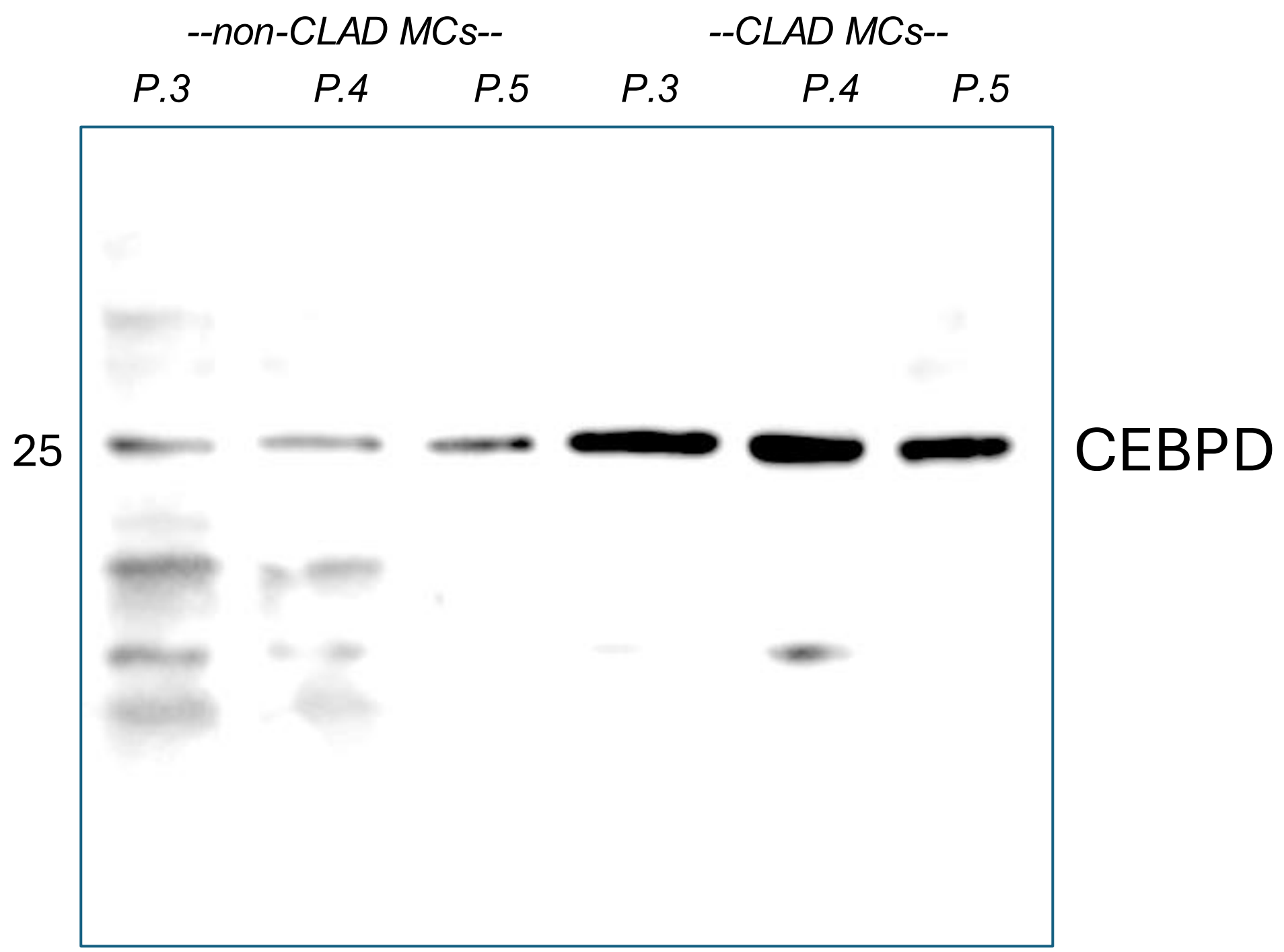

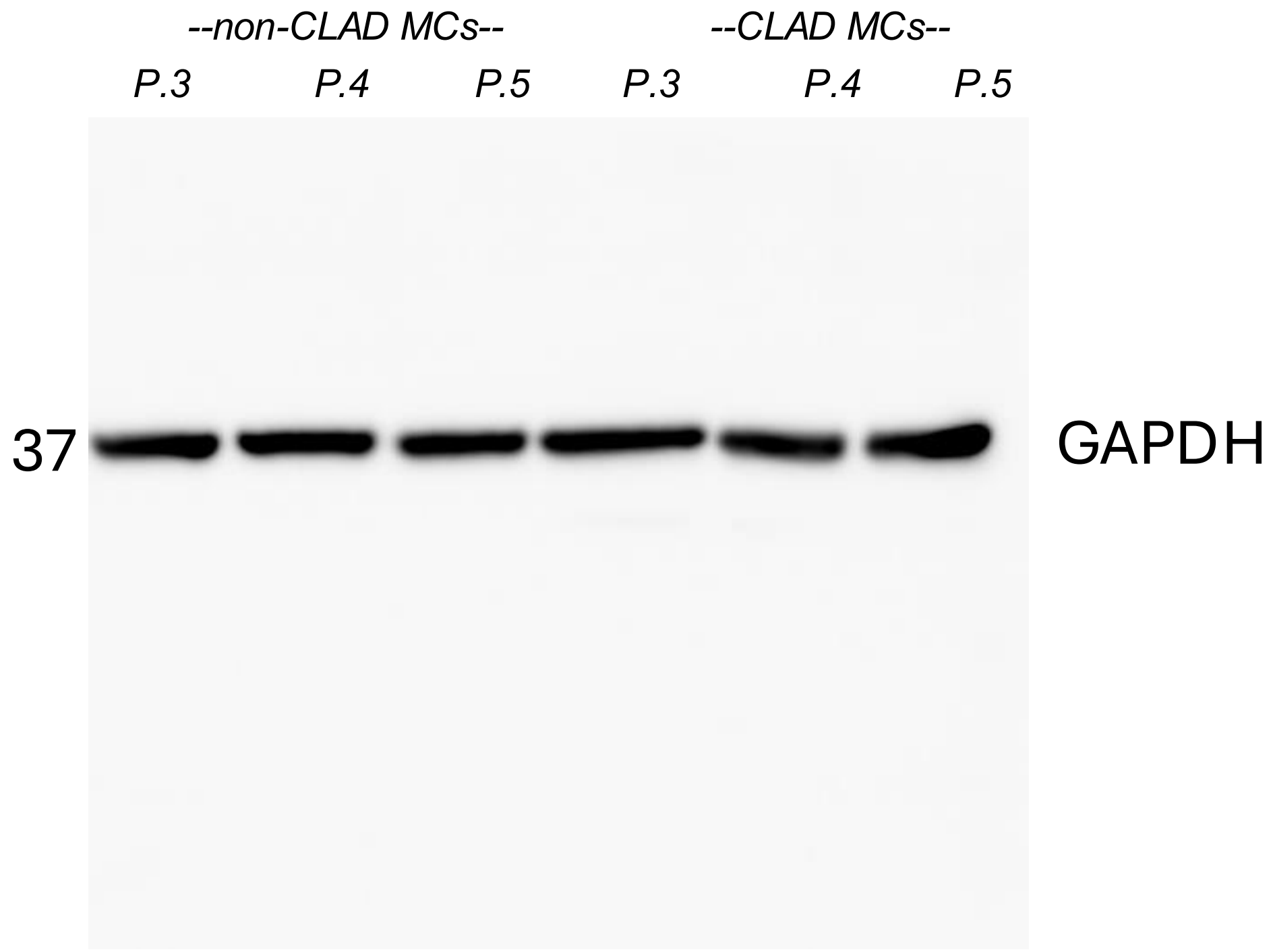

Supplement: Supplementary file 2 [file jciinsight-11-196651-s117.pdf]
